# Supplementary material for: Participant characteristics in the prevention of gestational diabetes as evidence for precision medicine: a systematic review and meta-analysis
Source: Commun Med (Lond). 2023 Oct 5;3:137. doi: 10.1038/s43856-023-00366-x (PMC10551015; doi:10.1038/s43856-023-00366-x)
Supplement: Supplementary file 1 — Supplmentary Data 1 [file 43856_2023_366_MOESM1_ESM.docx]

Supplementary Data 1. Summary characteristics of included studies

| First author, Year | Country | Sample size | Intervention commencement time (weeks) | Intervention type | Outcome of interest | GDM Diagnostic criteria | GDM as primary outcome |
| --- | --- | --- | --- | --- | --- | --- | --- |
| Randomised controlled trials | | | | | | |  |
| Abdel-Aziz, 2018 | Egypt | 160 | <12 | Diet + Physical activity | GDM | NR | No |
| Alamolhoda, 2019 | Iran | 800 | ≤7 | Diet | GDM | NR | Yes |
| Al wattar, 2018 | UK | 1138 | <18 | Diet | GDM/GWG | IADPSG* | Yes |
| Assaf- Balut, 2017 | Spain | 1000 | 8-12 | Diet | GDM/GWG | IADPSG | Yes |
| Barakat, 2012 | Spain | 83 | 6-9 | Physical activity | GDM/GWG | C&C | Yes |
| Barakat, 2013 | Spain | 428 | 10-12 | Physical activity | GDM/GWG | Other | Yes |
| Barakat, 2014 | Spain | 251 | 10-12 | Physical activity | GDM/GWG | NR | No |
| Barakat, 2019 | Spain | 456 | 8-10 | Physical activity | GDM/GWG | NR | Yes |
| Basu, 2021 | USA | 34 | <20 | Diet | GDM/GWG | ACOG** | No |
| Bogaerts, 2013 | Belgium | 197 | < 15 | Diet + Physical activity | GDM/GWG | Other | No |
| Bruno, 2016 | Italy | 131 | 9-12 | Diet + Physical activity | GDM/GWG | IADPSG | Yes |
| Buckingham-Schutt, 2019 | USA | 56 | 8–14 | Diet + Physical activity | GDM/GWG | IADPSG | No |
| Cahill 2018 | USA | 267 | ≤ 15 | Diet + Physical activity | GDM/GWG | IADPSG | No |
| Callaway, 2010 | Australia | 41 | 12 | Diet + Physical activity | GDM | ADIPS 1998 | No |
| Chan R, 2018 | China | 166 | ≤12 | Diet + Physical activity | GDM/GWG | IADPSG | Yes |
| Cordero, 2015 | Spain | 257 | 10 - 12 | Physical activity | GDM | NDDG | Yes |
| DaSilva, 2017 | Brazil | 612 | 16 - 20 | Physical activity | GDM/GWG | NR | No |
| Deng, 2022 | China | 84 | 14 | Diet + Physical activity |  | IADPSG | Yes |
| Ding 2021 | China | 215 | < 12 | Diet + Physical activity | GDM/GWG | IADPSG | Yes |
| Dodd 2019 | Australia | 633 | 16 | Diet + Physical activity | GDM/GWG | Other | No |
| Eslami 2018 | Iran | 140 | 16-20 | Diet + Physical activity | GDM | NR | Yes |
| Garmendia, 2020 | Chile | 4631 | <15 | Diet + Physical activity | GDM/GWG | Other | Yes |
| Gonzalez-Plaza, 2022 | Spain | 120 | 12-18 | Diet + Physical activity | GDM | IADPSG | No |
| Guelfi, 2016 | Australia | 169 | 14 | Physical activity | GDM/GWG | ADIPS 1991 | Yes |
| Hajian, 2020 | Iran | 66 | 16-20 | Diet + Physical activity | GDM/GWG | NR | No |
| Harrison, 2013 | Australia | 203 | 12-15 | Diet + Physical activity | GDM/GWG | Other | No |
| Herring, 2016 | USA | 56 | <12 | Diet + Physical activity | GDM/GWG | NR | No |
| Hui, 2006 | Germany | 45 | <26 | Diet + Physical activity | GDM/GWG | CDA 2008 | No |
| Hui, 2012 | Canada | 190 | <20 | Diet + Physical activity | GDM/GWG | CDA 2008 | No |
| Hui, 2014 | Germany | 113 | <20 | Diet + Physical activity | GDM/GWG | CDA 2008 | No |
| Huvinen, 2018, Rono, 2018, Koivusalo, 2016, Huvinen, 2022, Valkama, 2018, Grotenfelt, 2019 | Finland | 269 | <20 | Diet + Physical activity | GDM/GWG | C&C | Yes |
| Janumala, 2020 | USA | 187 | 14 | Diet + Physical activity | GDM/GWG | NR | No |
| Jing, 2015 | China | 221 | 12 | Diet + Physical activity | GDM/GWG | NR | No |
| Jovanovic-Peterson, 1997 | USA | 83 | NR | Diet | GDM | NDDG | No |
| Kennelly,2018 | Ireland | 498 | 10-15 | Diet + Physical activity | GDM/GWG | IADPSG | Yes |
| Ko,2012 | USA | 1116 | <20 | Physical activity | GDM | NR | No |
| Kong, 2014 | USA | 37 | 15 | Physical activity | GDM/GWG | NR | No |
| Korpi-Hyovalti, 2011 | Finland | 54 | 8-12 | Diet + Physical activity | GDM/GWG | Other | Yes |
| Kunath, 2019, Gunther, 2022, Hoffman, 2021 | Germany | 2286 | ≤12 | Diet + Physical activity | GDM/GWG | IADPSG | No |
| LeBlanc, 2020 | USA | 326 | Preconception | Diet + Physical activity | GDM/GWG | Other | No |
| Li, 2021 | China | 820 | NR | Diet + Physical activity | GDM/GWG | ACOG** | Yes |
| Lin, 2015 | China | 281 | < 8 | Diet + Physical activity | GDM | IADPSG | yes |
| Liu, 2015 | China | 296 | 8–12 | Diet + Physical activity | GDM/GWG | IADPSG | No |
| Liu, 2021 | USA | 217 | <18 | Diet + Physical activity | GDM/GWG | NR | No |
| Luoto, 2010 | Finland | 148 | <12 | Diet | GDM | Other | Yes |
| Luoto, 2011 | Finland | 1707 | 8–12 | Diet + Physical activity | GDM/GWG | C&C | Yes |
| McCarthy, 2016 | Australia | 148 | <20 | Diet | GDM | ADIPS 1998 | No |
| Mohsenzadeh-ledari F, 2020 | Iran | 109 | 15-20 | Diet + Physical activity | GDM/GWG | NR | Yes |
| Motahari-Tabari N, 2021 | Iran | 130 | 12 -17 | Diet + Physical activity | GDM | IADPSG | yes |
| Oostdam, 2012 | Netherlands | 99 | 15 | Physical activity | GDM/GWG | NR | No |
| Parat, 2019 | France | 267 | <21 | Diet + Physical activity | GDM | Other | No |
| Peccei, 2017 | USA | 255 | <16 | Diet + Physical activity | GDM/GWG | NR | No |
| Pelaez, 2019 | Spain | 301 | 12-36 | Physical activity | GDM/GWG | NDDG | No |
| Petrella, 2014 | Italy | 61 | 12 | Diet + Physical activity | GDM/GWG | IADPSG | No |
| Phelan, 2011 | USA | 349 | 10-16 | Diet + Physical activity | GDM/GWG | NR | No |
| Phelan, 2018 | USA | 257 | 9-16 | Diet + Physical activity | GDM/GWG | IADPSG | No |
| Phillips, 2019 | USA | 124 | <16 | Diet | GDM/GWG | NR | No |
| Polley, 2002 | USA | 110 | <20 | Diet + Physical activity | GDM/GWG | NR | No |
| Poston, 2015, Mills, 2019, Peacock, 2020 | UK | 1280 | 15-18+6 | Diet + Physical activity | GDM/GWG | IADPSG | Yes |
| Price, 2011 | USA | 62 | 12–14 | Physical activity | GDM/GWG | NR | No |
| Quinlivan, 2011 | Australia | 124 | NR | Diet | GDM/GWG | WHO 1999 | Yes |
| Rauh, 2013 | Germany | 235 | <20 | Diet + Physical activity | GDM/GWG | GSGO 2010 | No |
| Renault, 2014 | Denmark | 264 | 11-14 | Diet + Physical activity; physical activity | GDM | NR | No |
| Ruiz, 2013 | Spain | 962 | <12 | Physical activity | GDM/GWG | NR | No |
| Sagedal, 2017 | Norway | 557 | 20 | Diet + Physical activity | GDM/GWG | WHO 1999 | No |
| Sahariah, 2016 | India | 1008 | Preconception | Diet | GDM/GWG | WHO 1999 | Yes |
| Seneviratne, 2015 | New Zealand | 74 | <20 | Physical activity | GDM | Other | No |
| Simmons, 2017 | UK, Ireland, Netherlands, Austria, Poland, Italy, Spain, Denmark, Belgium | 192 | <20 | Diet + Physical activity; physical activity; diet | GDM | IADPSG | No |
| Stafne, 2012 | Norway | 702 | 18-22 | Physical activity | GDM/GWG | WHO 1999 | Yes |
| Sun, 2020 | China | 1162 | Preconception | Diet | GDM/GWG | NR | No |
| Thornton, 2009 | USA | 232 | 12-28 | Diet + Physical activity | GDM/GWG | NR | No |
| Tomić, 2013 | Croatia | 334 | 6-8 | Physical activity | GDM | NR | No |
| Trak-Fellermeier, 2019, Haslam, 2020 | USA | 31 | 14 | Diet + Physical activity | GDM | IADPSG | No |
| Van Horn, 2018 | USA | 260 | 16 | Diet + Physical activity | GDM/GWG | NR | No |
| Vesco, 2014 | USA | 114 | 8-21 | Diet + Physical activity | GDM | NR | No |
| Vinter, 2011 Vinter, 2014 | Denmark | 360 | 10-14 | Diet + Physical activity | GDM | Other | Yes |
| Walsh, 2012 | Ireland | 800 | ≤18 | Diet | GDM/GWG | C&C | No |
| Wang, 2015 | China | 299 | 8 | Diet + Physical activity | GDM/GWG | IADPSG | Yes |
| Wang, 2017 | China | 300 | 12+6 | Physical activity | GDM/GWG | IADPSG | Yes |
| Wolff, 2008 | Denmark | 50 | 15 | Diet | GDM/GWG | NR | No |
| Xu, 2022 | China | 348 | 12-24 | Diet + Physical activity | GDM/GWG | IADPSG | No |
| Zhang, 2015 | China | 261 | 12 | Diet | GDM | NR | Yes |
| Zhang, 2019 | China | 400 | <16 | Diet + Physical activity | GDM/GWG | IADPSG | Yes |
| Zhao, 2022 | China | 560 | 8-12 | Diet + Physical activity | GDM/GWG | IADPSG | Yes |
| Chiswick, 2008 | UK | 360 | 12-16 | Metformin | GDM/GWG | IADPSG | No |
| Dodd, 2018 | Australia | 524 | 16 | Metformin | GDM/GWG | Other | No |
| Jamal, 2012 | Iran | 105 | 12 | Metformin | GDM | NR | No |
| Lovik, 2019 | Norway | 487 | <12 | Metformin | GDM/GWG | WHO 1999 | No |
| Sales, 2018 | Brazil | 164 | ≤20 | Metformin | GDM | IADPSG | Yes |
| Syngelaki, 2016 | UK | 400 | 12-18 | Metformin | GDM | WHO 1999 | No |
| Valdés, 2018 | Spain | 111 | 12-15+6 | Metformin | GDM | IADPSG | Yes |
| Vanky, 2010 | Norway | 273 | 15 | Metformin | GDM | WHO 1999 | Yes |
| Callaway, 2019 | Australia | 207 | <20 | Probiotics | GDM/GWG | IADPSG | Yes |
| Celentano, 2020 | Italy | 157 | <12 | Myoinositol/  Inositol | GDM | IADPSG | Yes |
| D’Anna, 2015 | Italy | 220 | 12–13 | Myoinositol/  Inositol | GDM/GWG | IADPSG | Yes |
| D'Anna, 2013 | Italy | 220 | 12–13 | Myoinositol/  Inositol | GDM/GWG | IADPSG | Yes |
| Farren, 2017 | Ireland | 240 | 10-16 | Myoinositol/  Inositol | GDM | IADPSG | Yes |
| Lindsay, 2014 | Ireland | 175 | <20 | Probiotics | GDM/GWG | C&C | No |
| Luoto, 2010 | Finland | 146 | ≤12 | Diet+Probiotics; Diet | GDM | Other | Yes |
| Matarrelli, 2013 | Italy | 75 | 12 | Myoinositol/  Inositol | GDM | IADPSG | Yes |
| Pellonpera, 2019 | Finland | 190 | 18 | Fish oil; probiotics; fish oil+probioics | GDM | C&C | Yes |
| Santamaria, 2016 | Italy | 197 | 12-13 | Myoinositol/  Inositol | GDM/GWG | IADPSG | Yes |
| Vitale, 2021 | Italy | 223 | 12-13 | Myoinositol/  Inositol | GDM | IADPSG | Yes |
| Wickens, 2017 | New Zealand | 394 | 14-16 | Probiotics | GDM | IADPSG | Yes |
| Non Randomised Controlled Trials | | | | | | |  |
| Epel, 2019 | USA | 185 | 12-19 | Diet + Physical activity | GDM | NR | No |
| Gray-Donald, 2000 | Canada | 214 | <26 | Diet + Physical activity | GDM/GWG | NDDG | Yes |
| Gregory, 2016 | USA | 206 | <21 | Diet | GDM/GWG | NR | No |
| Liu, 2015 | USA | 54 | <18 | Diet + Physical activity | GDM/GWG | NR | No |
| Opie, 2016 | Australia | 217 | <21 | Diet | GDM/GWG | ADIPS 1991 | Yes |
| Shirazian, 2014 | USA | 120 | ≤14 | Diet + Physical activity | GDM/GWG | ACOG** | No |
| Shirazian, 2010 | USA | 41 | <12 | Diet + Physical activity | GDM/GWG | NR | No |
| Sun, 2016 | China | 66 | 8-12+6 | Diet + Physical activity | GDM/GWG | IADPSG | Yes |
| Adb El Hameed, 2011 | Egypt | 57 | Preconception | Metformin | GDM/GWG | C&C | Yes |
| Ainuddin, 2015 | Pakistan | 82 | Preconception | Metformin | GDM | WHO 1999 | Yes |
| Glueck, 2002a | USA | 72 | Preconception | Metformin | GDM | NDDG | Yes |
| Glueck, 2002b | USA | 72 | Preconception | Metformin | GDM | NDDG | No |
| Khattab, 2011 | Egypt | 360 | NR | Metformin | GDM | WHO 1999 | Yes |
