# Supplementary material for: Participant characteristics in the prevention of gestational diabetes as evidence for precision medicine: a systematic review and meta-analysis
Source: Commun Med (Lond). 2023 Oct 5;3:137. doi: 10.1038/s43856-023-00366-x (PMC10551015; doi:10.1038/s43856-023-00366-x)
Supplement: Supplementary file 3 — Supplmentary Data 3 [file 43856_2023_366_MOESM3_ESM.docx]

Supplementary Data 3. Meta-regression of interventions to prevent gestational diabetes, by participant characteristics

| Intervention and baseline covariate | Coefficient (95% CI) | P-value | Number of studies |
| --- | --- | --- | --- |
| Dietary intervention | | | |
| Age (years) | 0.01 (-0.07, 0.09) | 0.79 | 16 |
| BMI (kg/m^2^) | 0.04 (-0.01, 0.09) | 0.12 | 11 |
| Systolic blood pressure (mmHg) | 0.03 (-0.19, 0.25) | 0.67 | 4 |
| Fasting blood glucose (FBG) (mg/dL) | 0.12 (-1.4, 1.64) | 0.51 | 3 |
| Physical activity intervention | | | |
| Age (years) | 0.00 (-0.11, 0.12) | 0.93 | 19 |
| BMI (kg/m^2^) | 0.05 (-0.02, 0.11) | 0.13 | 17 |
| Systolic blood pressure (mmHg) | 0.10 (-1.31, 1.33) | 0.93 | 3 |
| HDL (mg/dl) | 0.00 (-0.22, 0.23) | 0.85 | 3 |
| LDL (mg/dl) | 0.00 (0.16, 0.17) | 0.82 | 3 |
| TG (mg/dl) | 0.00 (-0.09, 0.1) | 0.93 | 3 |
| Fasting blood glucose (FBG) (mg/dL) | -0.04 (-0.17, 0.08) | 0.38 | 6 |
| Combined (diet and physical activity) intervention | | | |
| Age (years) | -0.50 (-0.10, -0.03) | 0.03 | 55 |
| BMI (kg/m^2^) | -0.01 (-0.03, 0.01) | 0.47 | 50 |
| Systolic blood pressure (mmHg) | -0.01 (-0.06, 0.04) | 0.60 | 9 |
| HDL (mg/dl) | -0.01 (-0.01, 0.03) | 0.23 | 6 |
| LDL (mg/dl) | 0.01 (-0.01, 0.02) | 0.19 | 6 |
| TG (mg/dl) | -0.00 (-0.1, 0.00) | 0.23 | 6 |
| Fasting blood glucose (FBG) (mg/dL) | -0.00 (-0.01, 0.01) | 0.69 | 17 |
| Metformin intervention | | | |
| Age(years) | -0.52 (-1.02, -0.02) | 0.04 | 8 |
| BMI (kg/m^2^) | 0.12 (-0.12, 0.35) | 0.26 | 7 |
| Fasting blood glucose (FBG) (mg/dL) | -0.09 (-0.17, -0.03) | 0.02 | 7 |
| Myoinositol/inositol intervention | | | |
| Age(years) | 0.11(-0.39, 0.61) | 0.54 | 5 |
| BMI (kg/m^2^) | 0.11(-0.6, 0.83) | 0.69 | 6 |

BMI: body mass index, LDL: low-density lipoprotein, HDL: high-density lipoprotein, TG: triglyceride
