# Supplementary material for: Participant characteristics in the prevention of gestational diabetes as evidence for precision medicine: a systematic review and meta-analysis
Source: Commun Med (Lond). 2023 Oct 5;3:137. doi: 10.1038/s43856-023-00366-x (PMC10551015; doi:10.1038/s43856-023-00366-x)
Supplement: Supplementary file 4 — Supplmentary Data 4 [file 43856_2023_366_MOESM4_ESM.docx]

Supplementary Data 4. Subgroup analysis of the effect of physical activity interventions compared with control for gestational diabetes prevention, by participant characteristics

| Intervention type | The number of studies included | Risk ratio | Confidence interval | Heterogeneity (I^2^) (%) | p-value for subgroups | Weight |
| --- | --- | --- | --- | --- | --- | --- |
| Gestational week at baseline |  |  |  |  | 0.02 |  |
| Preconception | 0 | - | - | - | - | - |
| <12 gestation weeks | 7 | 0.51 | 0.34, 0.75 | 26.7 |  | 33.1 |
| 13-17 gestation weeks | 7 | 0.7 | 0.52, 0.96 | 16.7 |  | 37.9 |
| >18 gestation weeks | 5 | 1.02 | 0.75, 1.39 | 0 |  | 28.9 |
| BMI |  |  |  |  | <0.0001 |  |
| Normal weight | 1 | 0.06 | 0.03, 0.14 | - |  | 6 |
| Overweight/obese | 5 | 0.59 | 0.42, 0.81 | - |  | 22.7 |
| Obese | 3 | 0.8 | 0.44, 1.45 | 29.6 |  | 16.4 |
| All BMIs | 11 | 0.72 | 0.53, 0.97 | 36.8 |  | 54.9 |
| Educational status |  |  |  |  | 0.16 |  |
| With tertiary level education | 2 | 0.76 | 0.37, 0.1.53 | 77.5 |  | 41.5 |
| Without tertiary level education | 7 | 0.59 | 0.46, 0.77 | 0 |  | 36.8 |
| Unspecified | 10 | 0.88 | 0.66, 1.16 | 8.6 |  | 41.5 |
| Employment status |  |  |  |  | 0.87 |  |
| Employed | 7 | 0.69 | 0.51, 0.88 | 0 |  | 30.5 |
| Unemployed | 0 | - | - | - | - | - |
| Unspecified | 12 | 0.69 | 0.51, 0.94 | 48.2 |  | 65.9 |
| Hypertension at baseline |  |  |  |  | 0.25 |  |
| Without | 7 | 0.61 | 0.48, 0.77 | 0 |  | 37.9 |
| Unspecified | 12 | 0.77 | 0.57, 1.03 | 36.4 |  | 62.0 |
| Prediabetes at entry |  |  |  |  | 0.67 |  |
| Without | 9 | 0.67 | 0.49, 0.91 | 45.4 |  | 59.3 |
| Unspecified | 10 | 0.74 | 0.54, 1.01 | 14.1 |  | 40.7 |
| Parity |  |  |  |  | 0.31 |  |
| Nulliparous | 1 | 0.40 | 0.08, 2.10 | - |  | 1.4 |
| Not nulliparous | 1 | 0.12 | 0.02, 0.91 | - |  | 0.9 |
| Mixed | 12 | 0.75 | 0.55, 1.03 | 42.3 |  | 57.2 |
| Unspecified | 6 | 0.67 | 0.52, 0.87 | 0 |  | 35.9 |
| Ethnicity |  |  |  |  | 0.08 |  |
| White | 3 | 0.98 | 0.69, 1.41 | 16.2 |  | 23.9 |
| Non-white | - | - | - | - | - | - |
| Mixed | 5 | 0.80 | 0.53, 1.19 | 0 |  | 18.8 |
| Unspecified | 11 | 0.59 | 0.44, 0.79 | 30.2 |  | 57.4 |
| History of GDM |  |  |  |  | 0.09 |  |
| With | 1 | 1.01 | 0.70, 1.46 | - |  | 15.3 |
| Without | 3 | 0.42 | 0.18, 1.02 | 0 |  | 5.2 |
| Unspecified | 16 | 0.68 | 0.55, 0.89 | 24.1 |  | 81.9 |
| History of HDP |  |  |  |  | 0.72 |  |
| Without | 1 | 0.87 | 0.26, 2.91 | - |  | 2.7 |
| Unspecified | 18 | 0.69 | 0.56, 0.87 | 32.2 |  | 97.3 |

GDM: gestational diabetes, BMI: body mass index, HDP: hypertensive disorders of pregnancy
