# Supplementary material for: Participant characteristics in the prevention of gestational diabetes as evidence for precision medicine: a systematic review and meta-analysis
Source: Commun Med (Lond). 2023 Oct 5;3:137. doi: 10.1038/s43856-023-00366-x (PMC10551015; doi:10.1038/s43856-023-00366-x)
Supplement: Supplementary file 5 — Supplmentary Data 5 [file 43856_2023_366_MOESM5_ESM.docx]

Supplementary Data 5. Subgroup analysis of the effect of combined diet and physical activity interventions compared with control for gestational diabetes prevention, by participant characteristics

| Intervention type | The number of studies included | Risk ratio | Confidence interval | Heterogeneity (I^2^) (%) | p-value for subgroups | Weight |
| --- | --- | --- | --- | --- | --- | --- |
| Gestational week at baseline |  |  |  |  | 0.07 |  |
| Preconception | 1 | 0.71 | 0.44, 1.13 | - |  | 2.5 |
| <12 gestation weeks | 16 | 0.79 | 0.67, 0.93 | 31.4 |  | 31.3 |
| 13-17 gestation weeks | 23 | 0.87 | 0.72, 1.05 | 48.1 |  | 36.5 |
| >18 gestation weeks | 18 | 0.82 | 0.68, 0.99 | 41 |  | 28.7 |
| Trimester of  enrolment not  specified or across  more than one  trimester | 1 | 0.18 | 0.06, 0.52 | - |  | 0.85 |
| BMI |  |  |  |  | 0.04 |  |
| Normal weight | 2 | 1.66 | 0.72, 3.85 | 0 |  | 1.44 |
| Overweight/obese | 26 | 0.71 | 0.59, 0.87 | 57.3 |  | 43.5 |
| Obese | 12 | 0.94 | 0.80, 1.09 | 0 |  | 16.22 |
| All BMIs | 21 | 0.77 | 0.63, 0.95 | 68.7 |  | 38.8 |
| Educational status |  |  |  |  | 0.70 |  |
| With tertiary level education | 18 | 0.85 | 0.73, 0.99 | 14.6 |  | 30.9 |
| Without tertiary level education | 21 | 0.75 | 0.60, 0.95 | 63 |  | 32.2 |
| Unspecified | 20 | 0.83 | 0.70, 0.98 | 42.1 |  | 36.9 |
| Employment status |  |  |  |  | 0.08 |  |
| Employed | 14 | 0.74 | 0.58, 0.96 | 51.7 |  | 23.2 |
| Unemployed | 4 | 0.50 | 0.29, 0.85 | 37.9 |  | 4.9 |
| Unspecified | 41 | 0.88 | 0.78, 0.98 | 39.2 |  | 71.8 |
| Hypertension at baseline |  |  |  |  | 0.70 |  |
| Without | 13 | 0.78 | 0.62, 0.98 | 43.7 |  | 21.5 |
| Unspecified | 46 | 0.83 | 0.73, 0.94 | 48.8 |  | 78.5 |
| Prediabetes at entry |  |  |  |  | 0.14 |  |
| Without | 30 | 0.89 | 0.79, 0.99 | 26.0 |  | 54.02 |
| Unspecified | 29 | 0.75 | 0.62, 0.9 | 59.6 |  | 45.98 |
| Parity |  |  |  |  | 0.80 |  |
| Nulliparous | 2 | 1.02 | 0.27, 3.82 | 64.4 |  | 3.2 |
| Mixed | 38 | 0.82 | 0.72, 0.93 | 49.1 |  | 70.5 |
| Unspecified | 19 | 0.77 | 0.63, 0.93 | 27.1 |  | 26.3 |
| PCOS |  |  |  |  | 0.03 |  |
| With | 1 | 1.12 | 0.78, 1.61 | - |  | 3.1 |
| Without | 3 | 0.62 | 0.47, 0.82 | 0 |  | 6.8 |
| Unspecified | 55 | 0.83 | 0.74, 0.93 | 46 |  | 91 |
| Ethnicity |  |  |  |  | 0.61 |  |
| White | 6 | 0.83 | 0.65, 1.07 | 6.6 |  | 11 |
| Non-white | 5 | 0.83 | 0.58, 1.18 | 0 |  | 6.1 |
| Mixed | 15 | 0.92 | 0.76, 1.01 | 12.5 |  | 20.6 |
| Unspecified | 33 | 0.78 | 0.67, 0.91 | 61.5 |  | 62.3 |
| History of GDM |  |  |  |  | 0.03 |  |
| Without | 8 | 0.62 | 0.47, 0.81 | 20.5 |  | 12.1 |
| Unspecified | 51 | 0.85 | 0.76, 0.95 | 44.9 |  | 87.9 |

GDM: gestational diabetes, BMI: body mass index, PCOS: polycystic ovary syndrome
