# Supplementary material for: Participant characteristics in the prevention of gestational diabetes as evidence for precision medicine: a systematic review and meta-analysis
Source: Commun Med (Lond). 2023 Oct 5;3:137. doi: 10.1038/s43856-023-00366-x (PMC10551015; doi:10.1038/s43856-023-00366-x)
Supplement: Supplementary file 6 — Supplmentary Data 6 [file 43856_2023_366_MOESM6_ESM.docx]

Supplementary Data 6. Subgroup analysis of the effect of metformin interventions compared with control for gestational diabetes prevention, by participant characteristics

| Intervention type | The number of studies included | Risk ratio | Confidence interval | Heterogeneity (I^2^) (%) | p-value for subgroups | Weight |
| --- | --- | --- | --- | --- | --- | --- |
| Time at which the intervention was begun |  |  |  |  | <0.0001 |  |
| Preconception | 4 | 0.21 | 0.11, 0.40 | 0 |  | 15.8 |
| <12 gestation weeks | 2 | 0.96 | 0.58, 1.58 | 17.3 |  | 16.1 |
| 13-17 gestation weeks | 3 | 1.15 | 0.86, 1.55 | 0 |  | 30.1 |
| >18 gestation weeks | 3 | 0.93 | 0.65, 1.34 | 40.3 |  | 30.1 |
| Unspecified | 1 | 0.19 | 0.09, 0.34 | 0 |  | 7.9 |
| BMI |  |  |  |  | 0.37 |  |
| Overweight/obese | 3 | 0.37 | 0.09, 1.63 | 91.3 |  | 23.29 |
| Obese | 2 | 0.97 | 0.64, 1.48 | 0 |  | 18.5 |
| All BMIs | 8 | 0.75 | 0.51, 1.08 | 62.1 |  | 58.3 |
| Educational status |  |  |  |  | 0.08 |  |
| With tertiary level education | 1 | 1.06 | 0.78, 1.46 | - |  | 11.7 |
| Without tertiary level education | 1 | 0.81 | 0.42, 1.58 | - |  | 8.7 |
| Unspecified | 11 | 0.57 | 0.37, 0.89 | 76.9 |  | 79.7 |
| Employment status |  |  |  |  | 0.03 |  |
| Employed | 2 | 1.01 | 0.76, 1.34 | 0 |  | 20.33 |
| Unspecified | 11 | 0.57 | 0.37, 0.89 | 76.9 |  | 79.67 |
| Prediabetes at entry |  |  |  |  | 0.16 |  |
| Without | 5 | 0.89 | 0.57, 1.37 | 58.8 |  | 38.8 |
| Unspecified | 8 | 0.55 | 0.33, 0.92 | 78 |  | 61.2 |
| Parity |  |  |  |  | 0.02 |  |
| Mixed | 8 | 0.65 | 0.44, 0.97 | 77 |  | 76.2 |
| Unspecified | 5 | 0.37 | 0.18, 0.75 | 69 |  | 47.4 |
| PCOS |  |  |  |  | <0.001 |  |
| With | 8 | 0.38 | 0.19, 0.74 | 79.2 |  | 49.57 |
| Unspecified | 5 | 0.59 | 0.25, 1.43 | 5.6 |  | 50.4 |
| Ethnicity |  |  |  |  | 0.01 |  |
| White | 5 | 1.06 | 0.82, 1.39 | 38.3 |  | 45.08 |
| Mixed | 1 | 1.09 | 0.64, 1.88 | - |  | 9.78 |
| Unspecified | 7 | 0.39 | 0.21, 0.72 | 69.4 |  | 45.14 |
| History of GDM |  |  |  |  | 0.01 |  |
| Without | 2 | 1.20 | 0.85, 1.70 | 0 |  | 20.35 |
| Unspecified | 11 | 0.55 | 0.37, 0.84 | 75.7 |  | 79.65 |

GDM: gestational diabetes, BMI: body mass index, PCOS: polycystic ovary syndrome
