# Supplementary material for: Participant characteristics in the prevention of gestational diabetes as evidence for precision medicine: a systematic review and meta-analysis
Source: Commun Med (Lond). 2023 Oct 5;3:137. doi: 10.1038/s43856-023-00366-x (PMC10551015; doi:10.1038/s43856-023-00366-x)
Supplement: Supplementary file 7 — Supplmentary Data 7 [file 43856_2023_366_MOESM7_ESM.docx]

Supplementary Data 7. Subgroup analysis of the effect of myoinositol/inositol interventions compared with control for gestational diabetes prevention, by participant characteristics

| Intervention type | The number of studies included | Risk Ratio | Confidence interval | Heterogeneity (I) (%) | p-value for subgroups | Weight |
| --- | --- | --- | --- | --- | --- | --- |
| Gestational week at which the intervention was begun |  |  |  |  | 0.87 |  |
| Preconception | 1 | 0.40 | 0.27, 0.59 |  |  | 17.42 |
| <12 gestation weeks | 6 | 0.38 | 0.19, 0.74 |  |  | 82.6 |
| 13-17 gestation weeks | 0 | - | - | - | - | - |
| BMI |  |  |  |  | 0.88 |  |
| Normal weight | 1 | 0.42 | 0.24, 0.72 | - |  | 16.03 |
| Overweight/obese | 2 | 0.30 | 0.15, 0.59 | 54.2 |  | 29.6 |
| All BMIs | 2 | 0.35 | 0.02, 5.15 | 92.8 |  | 24.7 |
| Obese | 2 | 0.40 | 0.28, 0.58 | 0 |  | 29.6 |
| Hypertension |  |  |  |  | 0.82 |  |
| Without | 1 | 0.42 | 0.24, 0.72 | - |  | 83.9 |
| Unspecified | 6 | 0.38 | 0.2, 0.72 | 82.3 |  | 83.9 |
| Prediabetes at baseline |  |  |  |  | 0.07 |  |
| With | 1 | 0.08 | 0.02, 0.31 | - |  | 8.28 |
| Without | 5 | 0.46 | 0.24, 0.88 | 80.9 |  | 74.3 |
| Unspecified | 1 | 0.40 | 0.27, 0.59 | - |  | 17.2 |
| Ethnicity |  |  |  |  | <0.0001 |  |
| White | 2 | 0.41 | 0.25, 0.69 | 0 |  | 27.2 |
| Mixed | 1 | 1.27 | 0.77, 2.09 | - |  | 16.5 |
| Unspecified | 4 | 0.29 | 0.18, 0.48 | 60.2 |  | 56.4 |
| History of GDM |  |  |  |  | 0.81 |  |
| Without | 4 | 0.35 | 0.25, 0.49 | 1.5 |  | 57.85 |
| Unspecified | 3 | 0.41 | 0.13, 1.28 | 90.4 |  | 42.2 |

GDM: gestational diabetes, BMI: body mass index
