# Supplementary material for: Participant characteristics in the prevention of gestational diabetes as evidence for precision medicine: a systematic review and meta-analysis
Source: Commun Med (Lond). 2023 Oct 5;3:137. doi: 10.1038/s43856-023-00366-x (PMC10551015; doi:10.1038/s43856-023-00366-x)
Supplement: Supplementary file 8 — Supplementary Information [file 43856_2023_366_MOESM8_ESM.pdf]

Supplementary Table 1. Search strategies

Search Queries
**Medline (Ovid)**<br><br>1. Pregnancy/ or Gravidity/ or Preconception Care/<br>2. (antenatal or (ante* adj2 natal) or childbearing or (child adj2 bearing) or gestation* or family planning services or gravid* or interconcept* or intergestation* or internatal or matern* or periconcept* or preconcept* or pregestation* or prenatal* or prepregn* or ((inter or pre or peri) adj2 (concept* or gestation* or natal or pregnan*))).ti,ab,kw.<br>3. 1 or 2<br>4. Behavior Therapy/ or Cognitive Behavioral Therapy/ or Diet/ or Exercise/ or Health Behavior/ or Health Education/ or Health Promotion/ or Life Style/ or Weight Loss/<br>5. ((behav* adj2 (cognit* or manag* or modif* or therap*)) or CBT or diet* or (health* adj2 (behav* or eat* or educat* or food* or promotion)) or lifestyle or (life adj2 style) or nutrition or physical activit* or (weight adj2 (loss or manag* or reduction or retention))).ti,ab,kw.<br>6. 4 or 5<br>7. Biguanides/ or Metformin/ or Buformin/ or Chlorhexidine/ or Proguanil/ or Phenformin/<br>8. (biguanid* or metformin* or buformin* or chlorhexidin* or chlorguanid* or phenformin*).ti,ab,kw.<br>9. 7 or 8<br>10. 6 or 9<br>11. Blood Glucose/ or Diabetes, Gestational/ or Glucose Tolerance Test/ or Insulin/ or Insulin Resistance/ or Obesity/ or Overweight/<br>12. (gestational weight gain or glucose* or glycemic* or insulin resistan*).ti,ab,kw.<br>13. ((obes* or overweight or weight gain) adj3 (women or matern* or pregnan*)).ti,ab,kw.<br>14. 11 or 12 or 13<br>15. "Clinical Trials as Topic"/ or Controlled Clinical Trial/ or Randomized Controlled Trial/<br>16. (trial or randomiz* or RCT or randomis* or control group* or two arm* or (two adj2 arm) or quasiexperiment* or (quasi* adj2 experiment*) or (matched adj2 (cohort* or control*))).ti,ab.<br>17. randomi*.pt.<br>18. (intervention or assigned* or compar*).ti,ab.<br>19. 15 or 16 or 17 or 18<br>20. 3 and 10 and 14 and 19<br>21. 20 not (Animals/ not (Animals/ and Humans/))
**Embase**

#1. antenatal:ti,ab OR ((ante* NEAR/2 natal):ti,ab) OR childbearing:ti,ab OR ((child NEAR/2 bearing):ti,ab) OR gestation*:ti,ab OR 'family planning services':ti,ab OR gravid*:ti,ab OR interconcept*:ti,ab OR intergestation*:ti,ab OR internatal:ti,ab OR matern*:ti,ab OR periconcept*:ti,ab OR preconcept*:ti,ab OR pregestation*:ti,ab OR prenatal*:ti,ab OR prepregn*:ti,ab OR (((inter* OR pre* OR peri*)

NEAR/2 (concept* OR gestation* OR natal OR pregnan*)):ti,ab)

#2. 'pregnancy'/exp OR 'prepregnancy care'/exp

#3. #1 OR #2

#4. 'behavior therapy'/exp OR 'cognitive behavioral therapy'/exp OR 'diet'/exp OR 'exercise'/exp OR 'health behavior'/exp OR 'health education'/exp OR 'health promotion'/exp OR 'lifestyle'/exp OR 'body weight loss'/exp

#5 (((behav* NEAR/2 (cognit* OR manag* OR modif* OR therap*)):ti,ab) OR cbt:ti,ab OR diet*:ti,ab OR ((health* NEAR/2 (behav* OR eat* OR educat* OR food* OR promotion)):ti,ab) OR lifestyle:ti,ab OR ((life NEAR/2 style):ti,ab) OR nutrition:ti,ab OR physical:ti,ab) AND activit*:ti,ab OR ((weight NEAR/2 (loss OR manag* OR reduction OR retention)):ti,ab)

#6. #4 OR #5

#7. 'biguanide'/exp OR 'metformin'/exp OR 'buformin'/exp OR 'chlorhexidine'/exp OR 'proguanil'/exp OR 'phenformin'/exp

#8. biguanid*:ti,ab OR metformin*:ti,ab OR buformin*:ti,ab OR chlorhexidin*:ti,ab OR chlorguanid*:ti,ab OR phenformin*:ti,ab

#9. #7 OR #8

#10. #6 OR #9

#11. 'glucose blood level'/exp OR 'pregnancy diabetes mellitus'/exp OR 'glucose tolerance test'/exp OR 'insulin'/exp OR 'insulin resistance'/exp OR 'obesity'/exp

#12. 'gestational weight gain':ti,ab OR glucose*:ti,ab OR glycemic*:ti,ab OR glycaemic*:ti,ab OR ((insulin* NEAR/2 resist*):ti,ab)

#13. ((obes* OR overweight OR 'weight gain') NEAR/3 (women OR matern* OR pregnan*)):ti,ab

#14. #11 OR #12 OR #13

#15. 'crossover procedure':de OR 'double-blind

procedure':de OR 'randomized controlled trial':de OR 'single-blind procedure':de OR

random*:de,ab,ti OR factorial*:de,ab,ti OR crossover*:de,ab,ti OR ((cross NEXT/1 over*):de,ab,ti) OR placebo*:de,ab,ti OR ((doubl* NEAR/1 blind*):de,ab,ti) OR ((singl* NEAR/1 blind*):de,ab,ti) OR assign*:de,ab,ti OR allocat*:de,ab,ti OR volunteer*:de,ab,ti

#16. #3 AND #10 AND #14 AND #15 AND [humans]/lim

Pubmed

#1

("Pregnancy"[Mesh] OR "Gravidity"[Mesh] OR "Preconception Care"[Mesh]) OR (gravid*[Title/Abstract] OR pregnan*[Title/Abstract] OR childbearing[Title/Abstract] OR child bearing[Title/Abstract] OR gestation*[Title/Abstract] OR matern*[Title/Abstract] OR preconcept*[Title/Abstract] OR prenatal[Title/Abstract] OR antenatal[Title/Abstract] OR pre-concept*[Title/Abstract] OR "pre concept*"[Title/Abstract] OR prepregnan*[Title/Abstract] OR pre-pregnan*[Title/Abstract] OR "pre-pregnan*"[Title/Abstract] OR pregestation*[Title/Abstract] OR pre-gestation*[Title/Abstract] OR "pre gestation*"[Title/Abstract] OR periconcept*[Title/Abstract] OR peri-concept*[Title/Abstract] OR "peri concept*"[Title/Abstract] OR interconcept*[Title/Abstract] OR inter-concept*[Title/Abstract] OR "inter concept*"[Title/Abstract] OR interpregnan*[Title/Abstract] OR inter-pregnan*[Title/Abstract] OR "inter pregnan*"[Title/Abstract] OR intergestation*[Title/Abstract] OR inter-gestation*[Title/Abstract] OR "inter gestation*"[Title/Abstract] OR internatal[Title/Abstract] OR "family planning services"[Title/Abstract] OR "family-planning services"[Title/Abstract] OR ante-natal[Title/Abstract] OR pre-natal[Title/Abstract])

#2

(behavior therap*[Title/Abstract] OR behaviour therap*[Title/Abstract] OR behavioral therap*[Title/Abstract] OR behavioural therap*[Title/Abstract] OR behavior modif*[Title/Abstract] OR behaviour modif*[Title/Abstract] OR behavioral modif*[Title/Abstract] OR behavioural modif*[Title/Abstract] OR behavior manage*[Title/Abstract] OR behaviour manage*[Title/Abstract] OR behavioral manage*[Title/Abstract] OR behavioural manage*[Title/Abstract] OR cbt[Title/Abstract] OR diet*[Title/Abstract] OR health behavior*[Title/Abstract] OR health education[Title/Abstract] OR health promotion[Title/Abstract] OR healthy eat*[Title/Abstract] OR healthy food[Title/Abstract] OR life style[Title/Abstract] OR lifestyle[Title/Abstract] OR life-style[Title/Abstract] OR nutrition[Title/Abstract] OR physical activit*[Title/Abstract] OR weight loss[Title/Abstract] OR weight management[Title/Abstract] OR weight reduction[Title/Abstract] OR weight retention[Title/Abstract]) OR ("Behavior Therapy"[Mesh] OR "Cognitive Behavioral Therapy"[Mesh] OR "Diet"[Mesh] OR "Exercise"[mesh] OR "Health Behavior"[mesh] OR "Health Education"[Mesh] OR "Health Promotion"[Mesh] OR "Life Style"[Mesh] OR "Weight Loss"[Mesh])

#3

("Biguanides"[Mesh] OR "Metformin"[Mesh] OR "Buformin"[Mesh] OR "Chlorhexidine"[Mesh] OR "Proguanil"[Mesh] OR "Phenformin"[Mesh]) OR (biguanid*[Title/Abstract] OR metformin*[Title/Abstract] OR

buformin*[Title/Abstract] OR chlorhexidin*[Title/Abstract] OR chlorguanid*[Title/Abstract] OR phenformin*[Title/Abstract])

#4

#2 OR #3

#5

("Blood Glucose"[mesh] OR "Diabetes, Gestational"[Mesh] OR "Insulin"[mesh] OR "Insulin Resistance" OR "Glucose Tolerance Test"[mesh] OR "Obesity"[mesh] OR "Overweight"[mesh]) OR ("gestational weight gain"[Title/Abstract] OR "glucose intolerance" [Title/Abstract] OR glucose*[Title/Abstract] OR glycemic*[Title/Abstract] OR "insulin resistan*"[Title/Abstract] OR obesity[Title/Abstract])

#6

"Clinical Trials as Topic"[Mesh] OR "Randomized Controlled Trial"[Publication Type] OR trial[Title/Abstract] OR randomiz*[Title/Abstract] OR RCT[Title/Abstract] OR randomis*[Title/Abstract] OR "control group*"[Title/Abstract] OR "two-arm*"[Title/Abstract] OR "two arm"[Title/Abstract] OR "quasi-experiment*"[Title/Abstract] OR "matched cohort*"[Title/Abstract] OR "matched control*"[Title/Abstract] OR intervention[Title/Abstract] OR assigned*[Title/Abstract] OR compar*[Title/Abstract]

#7

#1 AND #4 AND #5 AND #6

Supplementary Table 2: Inclusion criteria

**Population**	**Intervention**	**Outcome**	**Limits**
All women (of childbearing age)	Interventions:<br>● Diet<br>● Physical activity<br>● Behavioral<br>● Lifestyle<br>● Combined (diet, behavioral, physical activity)<br>● Metformin<br>● Supplementation<br><br>Control:<br>● Usual care<br>● Placebo<br>● Minimal intervention (e.g. not more than 1 session a year	Primary<br>Gestational diabetes<br><br>Secondary<br>Gestational weight gain	Randomized or non-randomized controlled trials<br><br>Language: English<br><br>Years of publication: all years

Supplementary Table 3: Definition of participant characteristics

**Category**	**Definition**
Gestational age	● Preconception<br>● 1st trimester: ≤12 gestational week<br>● Early 2nd trimester: 13-17 weeks<br>● Late 2nd trimester: ≥18-26 weeks<br>(if it was a range, the highest number of the range was considered)
Educational status	● Tertiary education: if ≥50% of the participants had attended tertiary education.<br>● Not attended tertiary education: if <50% of the participants had attended tertiary education.
Employment	● Employed: if ≥50% of the participants were employed.<br>● Unemployed: if <50% of the participants were employed.
Ethnicity	● White: ≥80% of the participants were of European and other Caucasian origins.<br>● Non-white: if <80 were white.<br>● Mixed: a combination of White and non-White where neither was >80% for each group
BMI (based on inclusion criteria)	● Normal weight: if only participants in normal-weight BMI category were recruited.<br>● Overweight, obesity: if only participants in overweight/obesity BMI categories were recruited.
Parity	● Nulliparous: if all participants were nulliparous<br>● Not nulliparous: If none of the participants were nulliparous<br>● Mixed: A mixture of nulliparous and not nulliparous
Hypertension (based on inclusion criteria)	● With: if all participants had hypertension.<br>● Without: if all participants were free of hypertension.
Prediabetes (based on inclusion criteria)	● With: if all participants had prediabetes.<br>● Without: if all participants were free of prediabetes.

Polycystic ovary syndrome (PCOS) (based on inclusion criteria)	• With: if all participants had PCOS.<br>• Without: if all participants were free of PCOS.
Cardiovascular disease (CVD) (based on inclusion criteria)	• With: if all participants had CVD<br>• Without: if all participants were free of CVD
Smoking (based on inclusion criteria)	• With: if all participants had a history of smoking<br>• Without: if all participants were free of history of smoking
History of macrosomia (based on inclusion criteria)	• With: if all participants had a history of macrosomia<br>• Without: if all participants were free of history of macrosomia
History of gestational diabetes (GDM)(based on inclusion criteria)	• With: if all participants had a history of GDM<br>• Without: if all participants were free of history of GDM
History of hypertensive disorders during pregnancy (HDP) (based on inclusion criteria)	• With: if all participants had a history of HDP<br>• Without: if all participants were free of history of HDP
History of stillbirth (based on inclusion criteria)	• With: if all participants had a history of stillbirth<br>• Without: if all participants were free of history of stillbirth

Supplementary Table 4: Categorisation of participant characteristics for subgroup analysis

Author, year	Trimester	BMI	Tertiary educated	Employment	Ethnicity	Hypertension	Hyperlipidemia	Prediabetes	Parity	PCOS	Stillbirth	Family history o diabetes	Prior macrosomic infant	Past history of GDM	Past history of HDP	History of CVD	Smoking
Abdel-Aziz, 2018	1st	All BMIs	No	No	NR	NR	NR	NR	Nulliparous	NR	No	NR	NR	NR	NR	NR	NR
Adb El Hameed, 2011	Preconception	All BMIs	NR	NR	NR	NR	NR	NR	NR	Yes	NR	NR	NR	NR	NR	NR	NR
Ainuddin, 2015	Preconception	All BMIs	NR	NR	NR	NR	NR	NR	Mixed	Yes	NR	NR	NR	NR	NR	NR	NR
Alamolhoda, 2019	1st	All BMIs	No	No	NR	No	NR	No	NR	NR	No	No	No	No	No	No	No
Al Wattar, 2018	Early 2nd	All BMIs	NR	NR	Mixed	NR	NR	No	Mixed	NR	NR	NR	NR	No	NR	NR	NR
Assaf-Balut, 2017	1st	All BMIs	Yes	NR	Mixed	NR	NR	NR	Mixed	NR	NR	NR	NR	NR	NR	NR	NR
Barakat, 2013	1st	All BMIs	No	Yes	NR	No	NR	No	NR	NR	NR	NR	NR	NR	NR	NR	NR
Barakat, 2012	1st	All BMIs	No	Yes	NR	NR	NR	NR	Mixed	NR	NR	NR	NR	NR	NR	NR	NR
Barakat, 2019	1st	All BMIs	No	NR	NR	NR	NR	No	Mixed	NR	NR	NR	NR	No	NR	NR	NR
Barakat, 2014	1st	All BMIs	No	NR	NR	NR	NR	NR	Mixed	NR	NR	NR	NR	NR	No	No	NR
Basu, 2021	Late 2nd	Obese	NR	NR	Non-White	No	NR	NR	Mixed	NR	NR	NR	NR	NR	NR	NR	NR
Bogaerts, 2013	Early 2nd	Obese	No	Yes	Mixed	NR	NR	No	Mixed	NR	NR	NR	NR	NR	NR	NR	NR
Bruno, 2016	1st	Overweight/obese	No	Yes	White	No	NR	NR	Mixed	NR	NR	NR	NR	No	NR	NR	No
Buckingham-Schutt, 2019	Early 2nd	All BMIs	NR	NR	NR	No	NR	NR	NR	NR	NR	NR	NR	No	No	No	NR
Cahill, 2018	Early 2nd	Overweight/obese	No	NR	NR	NR	NR	No	Mixed	NR	NR	NR	No	No	NR	NR	No

Callaway, 2010	1st	Obese	NR	NR	NR	NR	NR	NR	NR	NR	NR	NR	NR	NR	NR	NR	NR
Callaway, 2019	Late 2nd	Overweight/obese	NR	NR	White	NR	NR	NR	Mixed	NR	NR	NR	NR	NR	NR	NR	NR
Celentano, 2010	1st	Obese	NR	NR	NR	NR	NR	NR	Mixed	NR	NR	NR	NR	NR	NR	NR	NR
Chan, 2018	1st	All BMIs	Yes	Yes	Non-White	NR	NR	No	Mixed	NR	NR	NR	NR	NR	NR	NR	No
Chiswick, 2008	Early 2nd	Overweight/obese	NR	NR	White	NR	NR	No	NR	NR	NR	NR	NR	No	No	NR	NR
Cordero, 2015	1st	All BMIs	NR	Yes	NR	NR	NR	NR	Mixed	NR	NR	NR	NR	NR	NR	NR	NR
D'Anna, 2013	Early 2nd	Obese	NR	NR	White	NR	NR	No	Mixed	NR	NR	Yes	NR	No	NR	NR	NR
D'Anna, 2015	Early 2nd	Nomal and overweight	NR	NR	NR	No	NR	No	Mixed	NR	NR	NR	NR	No	NR	NR	NR
Silva, 2017	Late 2nd	All BMIs	NR	Yes	Mixed	No	NR	No	NR	NR	NR	NR	NR	NR	NR	No	NR
Deng, 2022	Early 2nd	All BMIs	No	NR	NR	NR	NR	NR	NR	NR	NR	NR	NR	NR	NR	NR	NR
Ding, 2021	1st	Overweight/obese	NR	Yes	NR	NR	NR	No	Mixed	No	NR	No	No	No	NR	NR	NR
Dodd, 2018	Late 2nd	All BMIs	NR	NR	White	NR	NR	No	Mixed	NR	NR	NR	NR	NR	NR	NR	NR
Dodd, 2019	Late 2nd	Overweight/obese	NR	NR	Mixed	NR	NR	No	Mixed	NR	NR	NR	NR	NR	NR	NR	NR
Epel, 2019	Late 2nd	Overweight/obese	No	NR	Non-White	No	NR	No	NR	No	NR	NR	NR	NR	NR	No	NR
Eslami, 2018	Late 2nd	Overweight/obese	No	No	NR	NR	NR	NR	Mixed	NR	NR	NR	NR	No	NR	NR	NR
Farren, 2017	Early 2nd	All BMIs	NR	NR	Mixed	NR	NR	No	Mixed	NR	NR	Yes	NR	NR	NR	NR	NR
Garmendia, 2020	Early 2nd	All BMIs	No	NR	NR	NR	NR	NR	Mixed	NR	NR	NR	NR	NR	NR	NR	NR

Glueck, 2002	NR	All BMIs	NR	NR	White	NR	NR	No	NR	Yes	NR	NR	NR	NR	NR	NR	NR
Glueck, 2022	Preconception	Overweight/obese	NR	NR	NR	NR	NR	NR	Mixed	Yes	NR	NR	NR	NR	NR	NR	NR
Gonzalez-Plaza, 2022	Early 2nd	Obese	No	Yes	NR	No	NR	No	Mixed	NR	NR	NR	NR	NR	NR	No	NR
Gray-Donald, 2000	Late 2nd	All BMIs	NR	NR	NR	NR	NR	No	Mixed	NR	NR	NR	NR	NR	NR	NR	NR
Gregory, 2016	Early 2nd	Obese	NR	NR	Non-White	NR	NR	No	Mixed	NR	NR	NR	NR	NR	NR	No	NR
Hajian, 2020	Late 2nd	Overweight	No	NR	NR	NR	NR	NR	Mixed	NR	NR	NR	NR	NR	NR	NR	NR
Harrison, 2012	Early 2nd	Overweight/obese	Yes	Yes	NR	NR	NR	No	NR	NR	NR	NR	NR	NR	NR	NR	NR
Herring, 2016	1st	Overweight/obese	No	NR	Non-White	NR	NR	NR	Mixed	NR	NR	NR	NR	NR	NR	NR	NR
Hui, 2004	Late 2nd	All BMIs	No	NR	Mixed	NR	NR	No	NR	NR	NR	NR	NR	NR	NR	NR	NR
Hui, 2012	Late 2nd	All BMIs	NR	NR	Mixed	NR	NR	No	NR	NR	NR	NR	NR	NR	NR	NR	NR
Hui, 2014	Late 2nd	All BMIs	NR	NR	NR	NR	NR	NR	Mixed	NR	NR	NR	NR	NR	NR	NR	NR
Jamal, 2012	1st	All BMIs	NR	NR	NR	No	NR	No	Mixed	Yes	NR	NR	NR	NR	NR	NR	NR
Janumalal, 2020	Early 2nd	Overweight/obese	NR	NR	Mixed	NR	NR	NR	NR	NR	NR	NR	NR	NR	NR	NR	NR
Jing, 2015	1st	All BMIs	NR	NR	NR	NR	NR	No	NR	NR	NR	NR	NR	NR	NR	NR	NR
Jovanovic-Peterson, 1997	NR	All BMIs	NR	NR	Non-White	No	NR	NR	NR	NR	NR	NR	NR	NR	NR	NR	NR
Kennelly, 2018	Early 2nd	Overweight/obese	Yes	NR	White	NR	NR	NR	Mixed	NR	NR	NR	NR	NR	NR	NR	NR
Khattab, 2011	NR	Overweight/obese	NR	NR	NR	NR	NR	NR	Mixed	Yes	NR	NR	NR	NR	NR	NR	NR
Ko, 2012	Late 2nd	All BMIs	NR	NR	Mixed	NR	NR	NR	NR	NR	NR	NR	NR	NR	NR	NR	NR

Koivusalo 2016, Huvinen 2018, Rono, 2018, Huvinen 2022, Valkama 2018, Grotenfelt 2019, 2018	Late 2nd	All BMIs	No	NR	NR	NR	NR	NR	Mixed	NR	NR	NR	NR	NR	NR	NR	NR
Kong, 2014	Early 2nd	Overweight/obese	NR	Yes	NR	No	NR	No	NR	NR	NR	NR	NR	No	NR	No	No
Korpi-Hyovalti, 2011	1st	All BMIs	No	Yes	NR	NR	NR	NR	Mixed	NR	NR	NR	NR	NR	NR	NR	NR
Kunath 2019, Gunther 2022, Hoffman 2021, 2019	1st	All BMIs	No	NR	NR	NR	NR	No	Mixed	NR	NR	NR	NR	NR	NR	NR	NR
LeBlanc, 2020&2021	Preconception	Overweight/obese	Yes	NR	White	NR	NR	NR	NR	NR	NR	NR	NR	NR	NR	NR	NR
Li, 2021	NR	All BMIs	NR	NR	NR	NR	NR	NR	Mixed	NR	NR	NR	NR	NR	NR	NR	NR
Lin, 2020	1st	All BMIs	NR	NR	NR	NR	NR	NR	Mixed	NR	NR	NR	NR	NR	NR	NR	NR
Lindsay, 2014	Late 2nd	All BMIs	No	NR	White	NR	NR	No	Mixed	NR	NR	NR	NR	No	NR	NR	NR
Liu, 2015	1st	Overweight/obese	NR	NR	NR	No	NR	No	NR	Yes	NR	NR	NR	NR	NR	NR	NR
Liu, 2021	Early 2nd	Overweight/obese	Yes	Yes	Mixed	NR	NR	NR	Mixed	NR	NR	NR	NR	NR	NR	NR	NR
Liu, 2015	Early 2nd	Overweight/obese	No	Yes	NR	No	NR	No	NR	NR	NR	NR	NR	NR	NR	No	NR
Lovvik, 2019	1st	All BMIs	Yes	Yes	White	NR	NR	NR	Mixed	Yes	NR	NR	NR	NR	NR	NR	NR
Luoto, 2010	1st	All BMIs	Yes	NR	NR	NR	NR	NR	Mixed	NR	NR	NR	NR	NR	NR	NR	NR
Luoto, 2010	1st	All BMIs	Yes	NR	NR	NR	NR	NR	Mixed	NR	NR	NR	NR	NR	NR	NR	NR
Luoto and Kolu, 2011	1st	All BMIs	Yes	NR	NR	NR	NR	NR	Mixed	NR	NR	NR	NR	NR	NR	NR	NR

Matarrelli , 2013	Early 2nd	All BMIs	NR	NR	NR	NR	NR	Yes	Mixed	NR	NR	NR	NR	NR	NR	NR	NR
McCarthy, 2016	Late 2nd	Overweight/obese	Yes	NR	NR	NR	NR	NR	Mixed	NR	NR	NR	NR	NR	NR	NR	NR
Mohsenzadeh-ledari F, 2020	Late 2nd	All BMIs	No	No	NR	NR	NR	NR	Mixed	NR	NR	NR	NR	NR	NR	NR	NR
Motahari-Tabari N, 2021, 2021	Early 2nd	Overweight/obese	Yes	No	NR	NR	NR	No	Mixed	NR	NR	NR	NR	No	NR	NR	NR
Oostdam, 2012	Early 2nd	Obsese	No	Yes	Mixed	No	NR	NR	Mixed	NR	NR	NR	NR	NR	NR	NR	NR
Opie, 2016	Late 2nd	All BMIs	NR	NR	NR	NR	NR	No	Mixed	NR	NR	NR	NR	NR	NR	No	No
Parat, 2019	Late 2nd	Overweight/obese	Yes	NR	NR	NR	NR	No	Mixed	NR	NR	NR	NR	NR	NR	NR	NR
Peccei, 2017	1st	Overweight/obese	No	NR	Mixed	NR	NR	NR	NR	NR	NR	NR	NR	NR	NR	NR	NR
Pelaez, 2019	Early 2nd	Overweight/obese	NR	NR	NR	NR	NR	NR	NR	NR	NR	NR	NR	NR	NR	NR	NR
Pellonper¨a, 2019	1st	Overweight/obese	Yes	NR	White	NR	NR	NR	Mixed	NR	NR	NR	NR	NR	NR	NR	NR
Petrella, 2014	Early 2nd	Overweight/obese	No	Yes	Mixed	No	NR	No	Mixed	NR	NR	NR	NR	No	NR	NR	NR
Phelan, 2011	Early 2nd	All BMIs	Yes	NR	Mixed	NR	NR	NR	Mixed	NR	NR	NR	NR	NR	NR	NR	NR
Phelan, 2018	Early 2nd	Overweight/obese	Yes	NR	Mixed	NR	NR	No	Mixed	NR	NR	NR	NR	NR	NR	NR	NR
Phillips, 2019	Early 2nd	Overweight/obese	No	NR	White	NR	NR	NR	Mixed	NR	NR	NR	NR	NR	NR	NR	NR
Polley, 2002	Late 2nd	All BMIs	Yes	NR	Mixed	NR	NR	NR	NR	NR	NR	NR	NR	NR	NR	NR	NR
Poston 2015, Mills 2019, Peacock 2020, 2015	Late 2nd	Obese	NR	NR	Mixed	No	NR	No	Mixed	NR	NR	NR	NR	NR	NR	NR	NR
Price, 2011	Early 2nd	All BMIs	NR	NR	Mixed	No	NR	NR	Mixed	NR	NR	NR	NR	NR	NR	NR	NR
Quinlivan, 2011	NR	Overweight obese	NR	NR	Mixed	NR	NR	NR	Mixed	NR	NR	NR	NR	NR	NR	NR	NR

Rauh, 2013	Late 2nd	All BMIs	Yes	Yes	NR	NR	NR	NR	Mixed	NR	NR	NR	NR	NR	NR	NR	NR
Renault, 2014	Early 2nd	Obese	NR	NR	White	NR	NR	No	Mixed	NR	NR	NR	NR	NR	NR	NR	NR
Ruiz, 2013	1st	All BMIs	No	Yes	NR	NR	NR	NR	NR	NR	NR	NR	NR	NR	NR	NR	NR
Sagedal, 2016&17	Late 2nd	All BMIs	No	Yes	NR	NR	NR	No	Nulliparous	NR	NR	NR	NR	NR	NR	NR	NR
Sahariah , 2016	Preconception	All BMIs	No	No	Non-White	No	NR	NR	Mixed	NR	NR	NR	NR	NR	NR	NR	NR
Sales, 2018	Late 2nd	Obese	No	Yes	White	NR	NR	NR	NR	NR	NR	NR	NR	NR	NR	NR	NR
Santamaria, 2016	Early 2nd	Overweight	NR	NR	White	NR	NR	No	Mixed	NR	NR	NR	NR	No	NR	NR	NR
Seneviratne, 2015	Late 2nd	Overweight/obese	NR	Yes	Mixed	NR	NR	NR	Mixed	NR	NR	NR	NR	NR	NR	NR	No
Shirazian, 2010	Early 2nd	Obese	NR	NR	Non-White	No	NR	No	Mixed	NR	NR	NR	NR	NR	NR	NR	NR
Shirazian, 2016	Early 2nd	Obese	NR	NR	Non-White	No	NR	No	Mixed	NR	NR	NR	NR	NR	NR	NR	NR
Simmons, 2017	Late 2nd	Obese	Yes	NR	White	NR	NR	No	Mixed	NR	NR	NR	NR	NR	NR	NR	NR
Stafne, 2012	Late 2nd	All BMIs	NR	NR	NR	NR	NR	NR	Mixed	NR	NR	NR	NR	NR	NR	NR	NR
Sun, 2020	Preconception	All BMIs	No	Yes	Non-White	No	NR	No	NR	NR	NR	NR	NR	NR	NR	NR	NR
Sun, 2016	1st	Overweight/obese	Yes	NR	NR	NR	NR	NR	NR	NR	NR	NR	NR	NR	NR	NR	NR
Syngelaki, 2016	Early 2nd	Obese	NR	NR	Mixed	NR	NR	NR	Mixed	NR	NR	NR	NR	No	NR	NR	NR
Thornton, 2009	Late 2nd	Obese	NR	NR	Mixed	No	NR	NR	Mixed	NR	NR	NR	NR	NR	NR	NR	NR
Tomić, 2013	1st	All BMIs	No	NR	NR	No	NR	No	Mixed	NR	NR	NR	NR	NR	NR	No	NR

Trak-Fellermeier 2019, Haslam 2020, 2019	Early 2nd	Overweight/obese	Yes	NR	Mixed	NR	NR	No	Mixed	NR	NR	NR	NR	NR	NR	NR	No
Valdés, 2018	Early 2nd	All BMIs	NR	NR	NR	NR	NR	No	Mixed	NR	NR	NR	NR	NR	NR	NR	NR
Horn, 2018	Early 2nd	Overweight/obese	Yes	NR	Mixed	NR	NR	No	Mixed	NR	NR	NR	NR	NR	NR	NR	No
Vanky, 2010	Early 2nd	All BMIs	NR	NR	NR	NR	NR	NR	Mixed	Yes	NR	NR	NR	NR	NR	NR	NR
Vesco, 2014	Late 2nd	Obese	Yes	NR	White	NR	NR	No	NR	NR	NR	NR	NR	NR	NR	NR	NR
Vinter 2011 Vinter 2014, 2014	Early 2nd	Obese	Yes	Yes	NR	No	NR	No	Mixed	NR	NR	NR	NR	No	NR	No	NR
Vitale, 2021	Early 2nd	Overweight	NR	NR	NR	NR	NR	No	Mixed	NR	NR	NR	NR	No	NR	NR	NR
Walsh, 2012	Late 2nd	Normal weight	NR	NR	NR	NR	NR	NR	Not nulliparous	NR	NR	NR	Yes	No	NR	NR	NR
Wang, 2015	1st	All BMIs	NR	NR	NR	NR	NR	NR	NR	NR	NR	NR	NR	NR	NR	NR	NR
Wang, 2017	Early 2nd	Overweight	Yes	NR	NR	No	NR	No	Mixed	NR	NR	NR	NR	NR	NR	No	No
Wickens, 2017	Early 2nd	All BMIs	NR	NR	Mixed	NR	NR	NR	Mixed	NR	NR	NR	NR	NR	NR	NR	NR
Wolff, 2008	Early 2nd	Obese	NR	NR	White	NR	NR	NR	NR	NR	NR	NR	NR	NR	NR	NR	No
Xu, 2022	Late 2nd	All BMIs	NR	NR	NR	NR	NR	NR	Mixed	NR	NR	NR	NR	NR	NR	NR	NR
Zhang, 2015	1st	All BMIs	NR	NR	NR	NR	NR	NR	NR	NR	NR	NR	NR	NR	NR	NR	NR
Zhang, 2019	Early 2nd	Overweight/obese	NR	NR	NR	No	NR	No	NR	NR	NR	NR	NR	NR	NR	No	NR
Zhao, 2022	1st	All BMIs	No	Yes	NR	NR	NR	NR	Mixed	No	NR	NR	NR	NR	NR	NR	NR

BMI: Body mass index, PCOS: polycystic ovary syndrome; GDM: Gestational diabetes; HDP: Hypertensive disorder during pregnancy; CVD: cardiovascular disease; NR: Not reported; Yes: With condition; No: Without condition;

Supplementary Table 5: Risk of bias summary

Supplementary Table 5a: Risk of bias for RCTs

Author, Year	Randomization process	Deviations from intended interventions	Missing outcome data	Measurement of the outcome	Selection of the reported result	Overall Bias
**Intervention type: Physical activity-only**						
Barakat, 2013	Low	Low	Low	Low	Low	Some concerns
Barakat, 2014	Some concerns	High	Low	Low	Low	High
Barakat, 2019	Low	Some concerns	Low	Low	Low	Some concerns
Basu, 2021	Some concerns	High	Low	Low	Low	Some concerns
Cordero, 2015	Some concerns	Low	Low	Low	Some concerns	Some concerns
DaSilva, 2017	Low	High	High	Low	Low	High
Guelfi, 2016	Low	Low	Low	Low	Some concerns	Some concerns
Ko, 2012	Some concerns	Some concerns	Low	Low	Some concerns	Some concerns
Kong, 2014	Low	Low	Low	Some concerns	Some concerns	Some concerns
Oostdam, 2012	Low	High	High	Low	Low	High
Pelaez, 2019	Some concerns	Low	Low	Low	Low	Some concerns
Price, 2011	Low	Low	High	Low	Low	High
Ruiz, 2013	Some concerns	Low	Low	Low	Low	Some concerns
Seneviratne, 2015	Low	Some concerns	Low	Low	Low	Some concerns
Stafne, 2012	Low	Low	Low	Low	Low	Low
Wang, 2017	Low	Some concerns	Low	Low	Low	Some concerns
**Intervention type: Diet-only**						
Alamolhoda, 2019	Some concerns	Low	Low	Low	Low	Some concerns
Alwattar, 2018	Some concerns	High	Low	Low	Some concerns	High
Assaf, 2017	High	High	High	Low	Low	High
Bogaerts, 2013	Low	Low	Low	Low	Low	Low
Jovanovic-Peterson, 1997	Some concerns	Some concerns	Low	Low	Some concerns	Some concerns
Luoto, 2010	Low	Low	High	Some concerns	Low	Some concerns
McCarthy, 2016	Low	Low	Low	Low	Low	Low
Phillips, 2019	Some concerns	Low	High	Low	Low	High
Quinlivan, 2011	Low	Low	Low	Low	Some concerns	Some concerns
Sahariah, 2016	Low	Low	High	Low	Low	High
Sun, 2020	Low	Low	Low	Some concerns	High	High

Walsh, 2012	Low	Some concerns	Low	Low	Low	Some concerns
Wolff, 2008	Low	Low	Low	Low	Low	Low
Zhang, 2015	High	High	High	Some concerns	Some concerns	High
**Intervention type: Combined (physical activity and diet).**						
Abdel-Aziz, 2018	Some concerns	Low	Low	Low	Low	Some concerns
Barakat, 2012	Some concerns	High	Low	Low	Low	High
Bruno, 2016	Low	Low	Low	Low	Low	Low
Buckingham-Schutt 2019	Some concerns	High	Low	Low	Low	High
Cahill, 2018	High	Low	Low	Low	Low	High
Callaway, 2010	Some concerns	High	High	Low	Low	High
Chan R, 2018	Low	Low	Low	Low	Low	Low
Deng, 2022	High	Low	Low	Low	Low	High
Ding, 2021	Low	Low	Low	Some concerns	Low	Some concerns
Dodd, 2019	Low	Low	Low	Low	Low	Low
Eslami, 2018	Some concerns	Some concerns	Low	Some concerns	Low	Some concerns
Gonzalez-Plaza, 2022	Low	Some concerns	Low	Low	Some concerns	Some concerns
Harrison, 2013	Low	Low	Low	Low	Some concerns	Some concerns
Herring, 2016	Low	Low	Low	Low	Some concerns	Some concerns
Hui, 2006	Some concerns	Some concerns	Low	Low	Some concerns	Some concerns
Hui, 2012	Low	Some concerns	Low	Low	Low	Some concerns
Hui, 2014	Low	Some concerns	Low	Low	Low	Some concerns
Janumala, 2020	Some concerns	High	Low	Low	Some concerns	High
Jing, 2015	Low	Some concerns	Low	Low	Low	Some concerns
Kennelly, 2018	Low	Some concerns	Low	Low	Low	Some concerns
Huvinen 2018,Rono 2018, Koivusalo 2016, Huvinen 2022, Valkama 2018, Grotenfelt 2019	Low	Some concerns	Low	Low	Some concerns	Some concerns
Korpi-Hyovalti, 2011	Low	Some concerns	Low	Low	Some concerns	Some concerns
LeBlanc, 2020	Low	Some concerns	Low	Low	Some concerns	Some concerns
Li, 2021	Low	Some concerns	Low	Low	Some concerns	Some concerns
Lin, 2020	Low	Some concerns	Low	Low	Some concerns	Some concerns
Liu, 2015	Low	Some concerns	Some concerns	Low	Low	Some concerns

Liu, 2021	Low	Some concerns	Low	High	Some concerns	High
Mohsenzadeh-ledari F, 2020	Some concerns	Low	Low	Low	Some concerns	Low
Motahari-Tabari N, 2021	Low	Some concerns	Low	Low	Low	Some concerns
Parat, 2019	Low	High	Low	Low	Low	High
Peccei, 2017	Some concerns	High	Low	Low	Low	High
Petrella, 2014	Some concerns	High	Low	Low	Low	High
Phelan, 2011	Low	Some concerns	Low	Low	Low	Some concerns
Phelan, 2018	Some concerns	Some concerns	Low	Low	Low	Some concerns
Polley, 2002	Some concerns	High	High	Low	Low	High
Poston 2015, Mills, 2019, Peacock, 2020	Some concerns	Low	High	Low	Low	High
Renault, 2014	Low	High	High	High	Low	High
Sagedal, 2017	Low	Low	Low	Low	Low	Low
Simmons, 2017	Low	Low	Low	Low	Low	Low
Thornton, 2009	Low	Low	Low	Low	Some concerns	Some concerns
Trak-Fellermeier, 2019, Haslam, 2020	Low	Low	Low	Low	High	Some concerns
Van Horn, 2018	Low	Low	Low	Low	Some concerns	Some concerns
Vesco, 2014	Low	Some concerns	Low	Low	Low	Low
Vinter, 2011, Vinter, 2014	Low	Low	Low	Low	Low	Low
Xu, 2022	Low	High	Low	Low	Low	Some concerns
Zhang, 2019	Low	Low	Low	Low	Low	Low
Zhao, 2022	Low	Low	Low	Low	Low	Low
**Intervention type: Myoinositol/inositol**						
D’Anna, 2015	Some concerns	Some concerns	Low	Low	Low	Some concerns
D'Anna, 2013	Low	Low	Low	Low	Low	Low
Matarrelli, 2013	Low	Low	Low	Low	Low	Low
Santamaria, 2015	Some concerns	High	High	Low	Low	High
Vitale, 2021	Low	High	Low	Low	Low	High
Celentano, 2020	Some concerns	Low	Low	Low	Low	Some concerns
Farren, 2017	Low	High	Low	Low	Low	High
**Intervention type: probiotics**						
Callaway, 2019	Low	Low	Low	Low	Low	Low
Lindsay, 2014	Some concerns	High	Low	Low	Low	High
Wickens, 2017	Low	Low	Low	Low	Low	Low
Luoto, 2021	Low	Low	Low	Low	High	High

Pellonpera, 2019	High	Low	Low	Low	Low	High
**Intervention type: metformin**						
Chiswick, 2008	Low	Low	Low	Low	Low	Low
Dodd, 2018	Low	Low	Low	Low	Low	Low
Jamal, 2012	Low	Some concerns	Low	Low	Low	Some concerns
Sales, 2018	Some concerns	High	Low	Low	Low	High
Syngelaki, 2016	Low	Low	Low	Low	Low	Low
Valdés 2018	Low	High	Low	Low	High	High
Vanky 2010	Low	Low	Low	Low	Some concerns	Some concerns

Supplementary Table 5b: Risk of bias for cluster-RCTs

Author, Year	Randomization process	Identification of recruitment of participants	Deviations from intended interventions	Missing outcome data	Measurement of the outcome	Selection of the reported result	Overall Bias
**Intervention type: Combined (diet and physical activity)**							
Garmendia, 2020	High	Low	Some concerns	Low	Low	Low	High
Hajian, 2020	Some concerns	Low	Low	Low	Low	Some concerns	Some concerns
Kunath, 2019, Gunther, 2022, Hoffman, 2021	Low	Low	Low	Low	Some concerns	Some concerns	Some concerns
Luoto, 2011	Low	High risk	Low risk	Low	Low risk	Low risk	High
Rauh, 2013	High	Low	Low	Low	Low	Low	High
Wang, 2015	Low	Some concerns	Low	Low	Low	Low	Some concerns
**Intervention type: Metformin**							
Lovvik, 2019	Low	Low	High	Low	Low	Some concerns	Some concerns

Supplementary Table 5c: Risk of bias for non-RCTs.

Author, Year	Confounding	Selection of participants	Classification of interventions	Deviations from intended interventions	Missing data	Measurement of outcomes	Selection of the reported result	Overall Bias
**Intervention type: Diet-only**								
Opie, 2016	Critical	Low	Low	Critical	Low	Moderate	Low	Critical
Gregory, 2016	Moderate	Low	Moderate	NI	Low	Low	Low	Moderate
**Intervention type: Combined (Diet and physical activity)**								
Epel, 2019	Critical	Low	Low	Critical	Low	Low	Low	Critical
Gray-Donald, 2000	Serious	Low	Low	NI	Low	Low	Low	Serious
Liu, 2015	Moderate	Low	Low	Moderate	Low	Moderate	Low	Serious
Shirazian, 2010	Low	Low	Low	Low	Moderate	Low	Low	Moderate
Shirazian, 2014	Low	Low	Low	Serious	Low	Low	Low	Serious
Sun, 2016	Low	Low	Low	NI	Low	Low	Low	Low
**Intervention type: Metformin**								
Abd El Hameed, 2011	Low	Low	Low	Moderate	Serious	Moderate	Low	Moderate
Ainuddin, 2015	Serious	Moderate	Low	NI	Low	Low	Moderate	Serious
Glueck, 2002a	Critical	Low	Low	Critical	Critical	Moderate	Low	Critical

Glueck, 2002	Serious	Low	Low	NI	Low	Low	Moderate	Serious
Khattab, 2011	Serious	Low	Serious	NI	NI	Low	Moderate	Serious
**Intervention type: physical activity-only**								
Tomic, 2013	Critical	Low	Moderate	Low	Moderate	Moderate	Low	Critical

Note: NI=No information; the order of severity of bias is Low<Moderate<Serious<Critical

Supplementary Table 5d: Summary of risk of bias assessment for RCTs

	Randomization process	Deviations from intended interventions	Missing outcome data	Measurement of the outcome	Selection of the reported result	Overall Bias
**Risk classification**	Low=63	Low=47	Low=82	Low=88	Low=69	Low=21
	Some concerns=28	Some concerns=26	Some concerns=1	Some concerns=6	Some concerns=23	Some concerns=44
	High=5	High=23	High=13	High=2	High=4	High=30

Supplementary Table 5e: Summary of risk of bias assessment for cluster RCTs

	Randomization process	Identification of recruitment of participants	Deviations from intended interventions	Missing outcome data	Measurement of the outcome	Selection of the reported result	Overall Bias
**Risk classification**	Low=4	Low=5	Low=5	Low=7	Low=6	Low=4	Low=0
	Some concerns=1	Some concerns=1	Some concerns=1	Some concerns=0	Some concerns=1	Some concerns=3	Some concerns=4
	High=2	High=1	High=1	High=0	High=0	High=0	High=3

Supplementary Table 5g: Summary of risk of bias assessment for non-RCTs

	Confounding	Selection of participants	Classification of interventions	Deviations from intended interventions	Missing data	Measurement of outcomes	Selection of the reported result	Overall Bias
**Risk classification**	Low=4	Low=13	Low=11	Low=2	Low=9	Low=9	Low=11	Low=1
	Moderate=2	Moderate =1	Moderate=2	Moderate=2	Moderate=2	Moderate =5	Moderate= 3	Moderate=3
	Critical= 4	Critical=0	Critical= 0	Critical= 3	Critical= 1	Critical= 0	Critical=0	Critical=4
	Serious= 4	Serious=0	Serious= 1	Serious= 1	Serious= 1	Serious= 0	Serious=0	Serious=6
	No information=0	No information =0	No information =0	No information =1	No information =1	No information =0	No information =0	No information =0

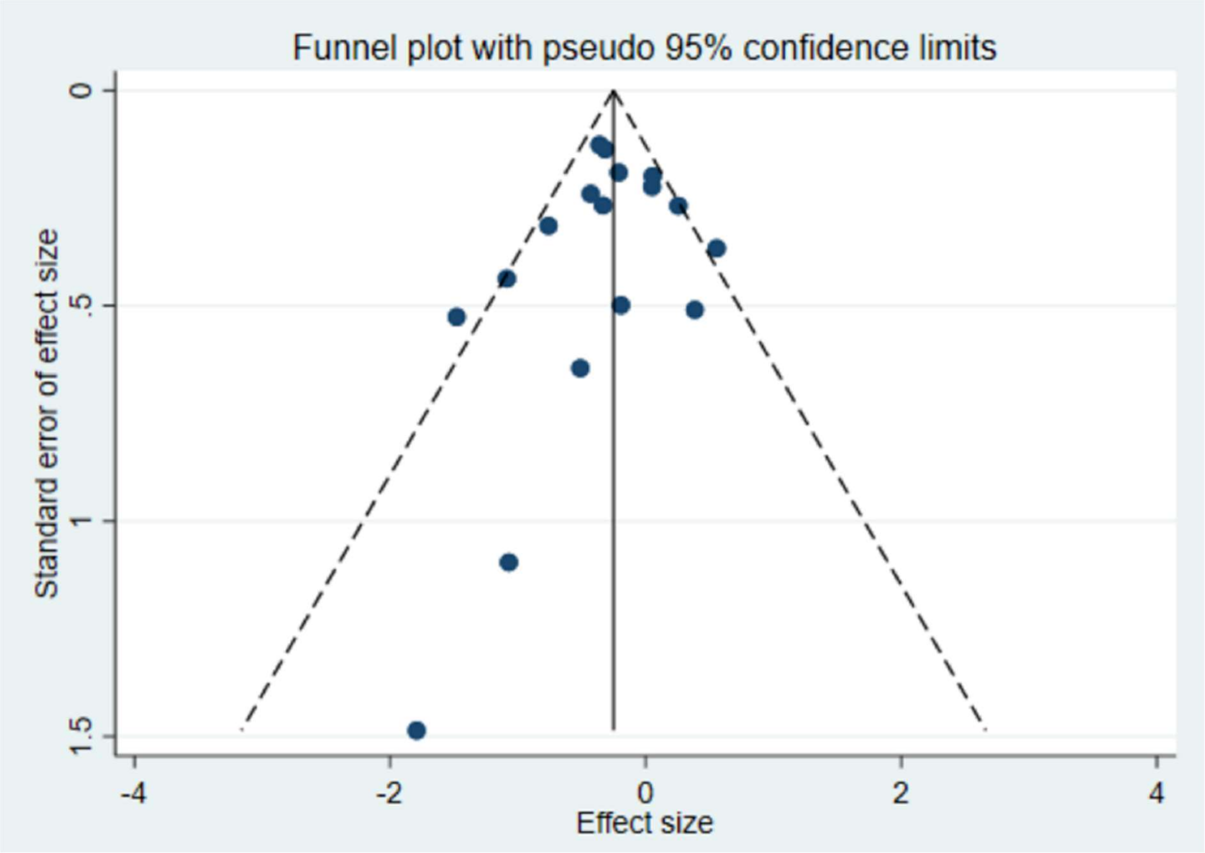

Supplementary Figure 1: A funnel plot showing the publication bias of studies on metformin intervention for preventing GDM.

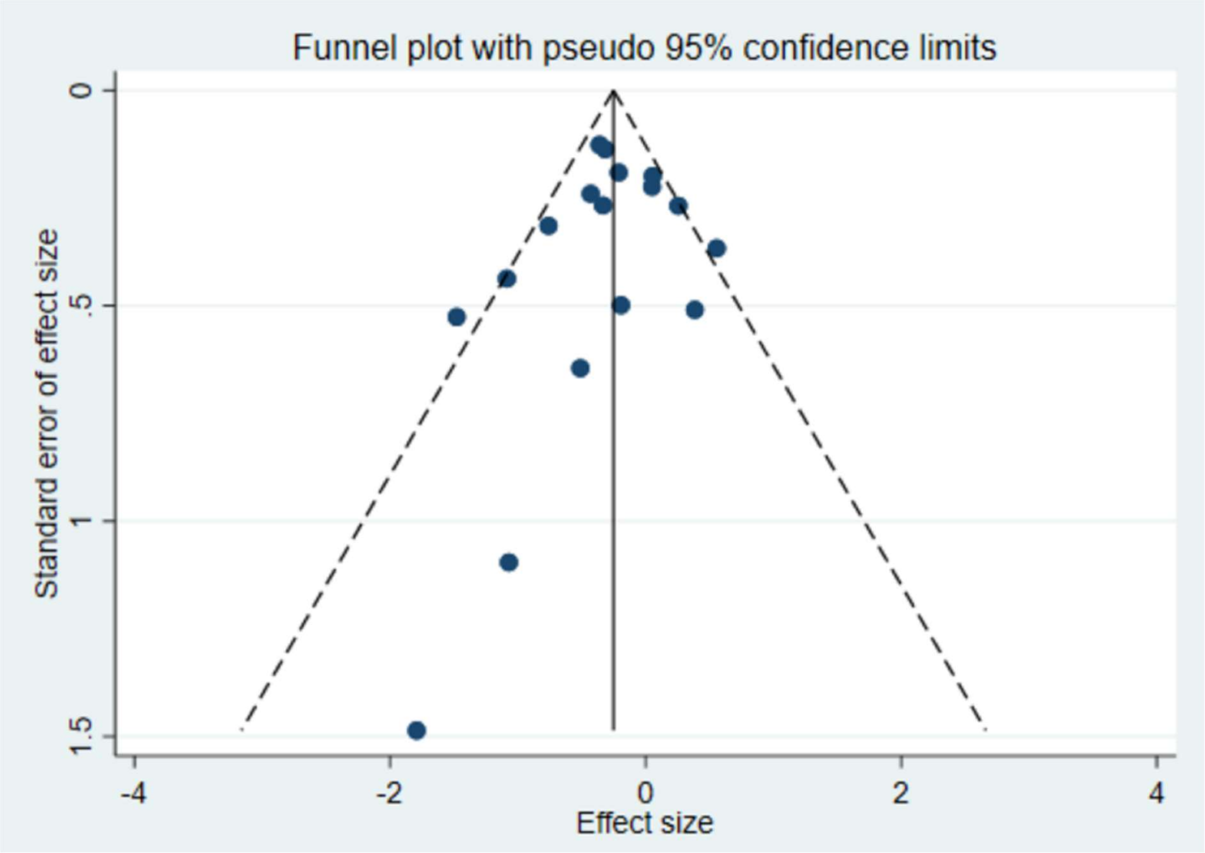

Supplementary Figure 2: A funnel plot showing the publication bias of studies on dietary interventions for preventing GDM.

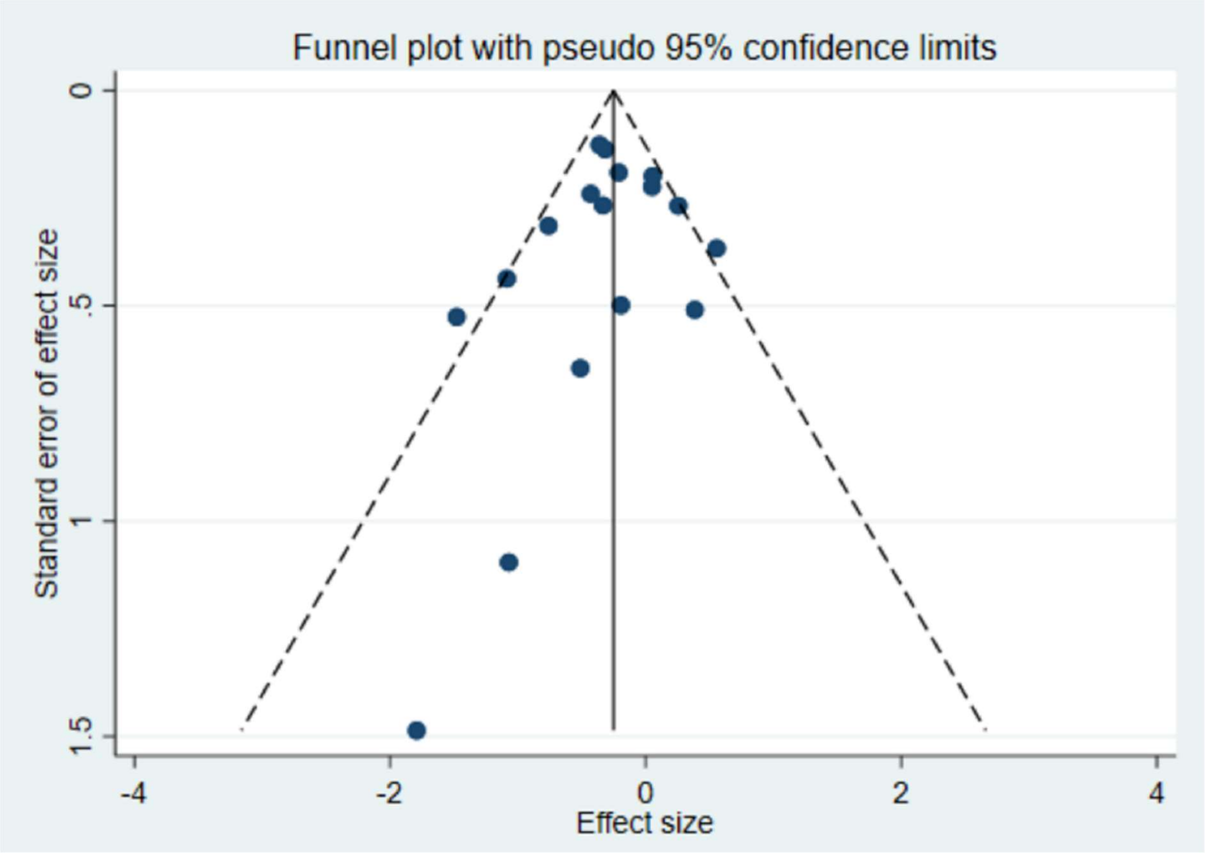

Supplementary Figure 3: A funnel plot showing the publication bias of studies on physical activity interventions for preventing GDM.

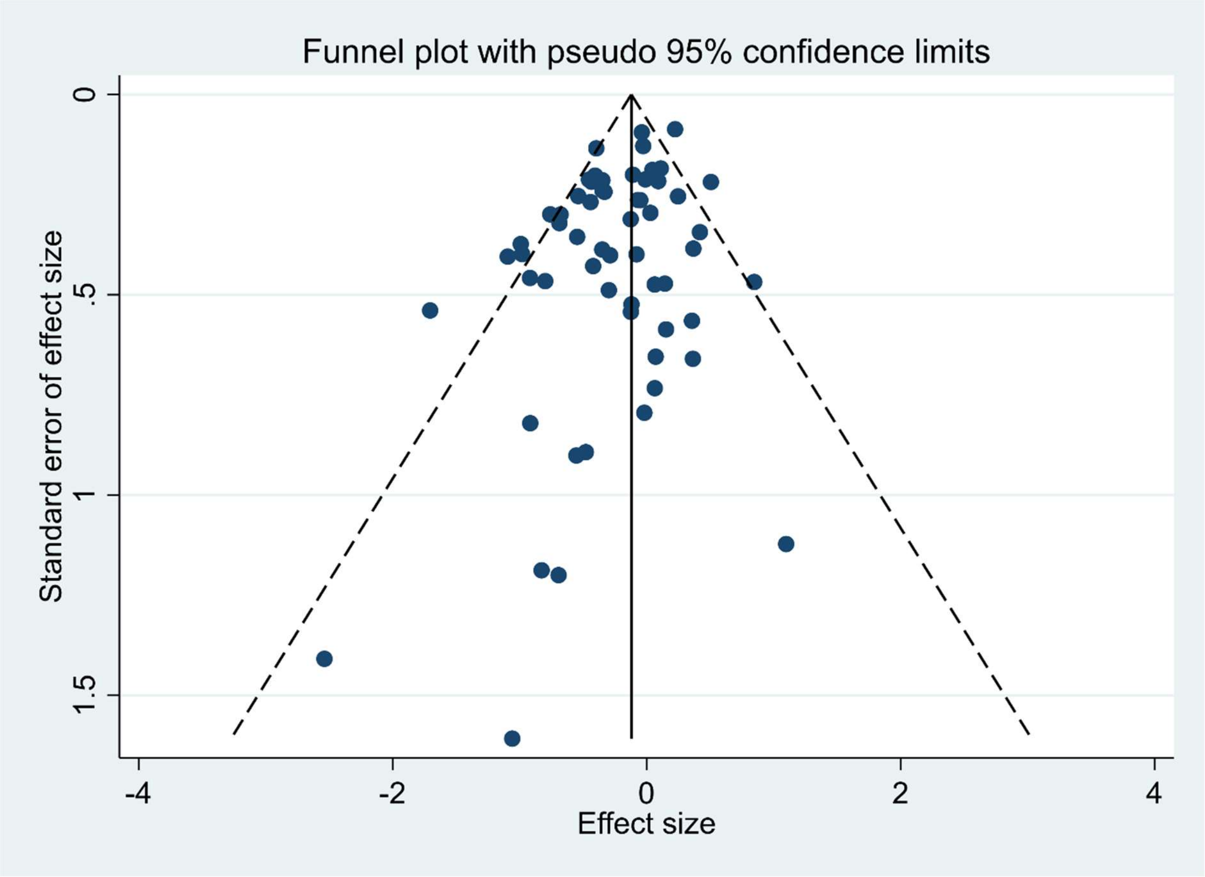

Supplementary Figure 4: A funnel plot showing the publication bias of studies on combined dietary and physical activity interventions for preventing GDM.

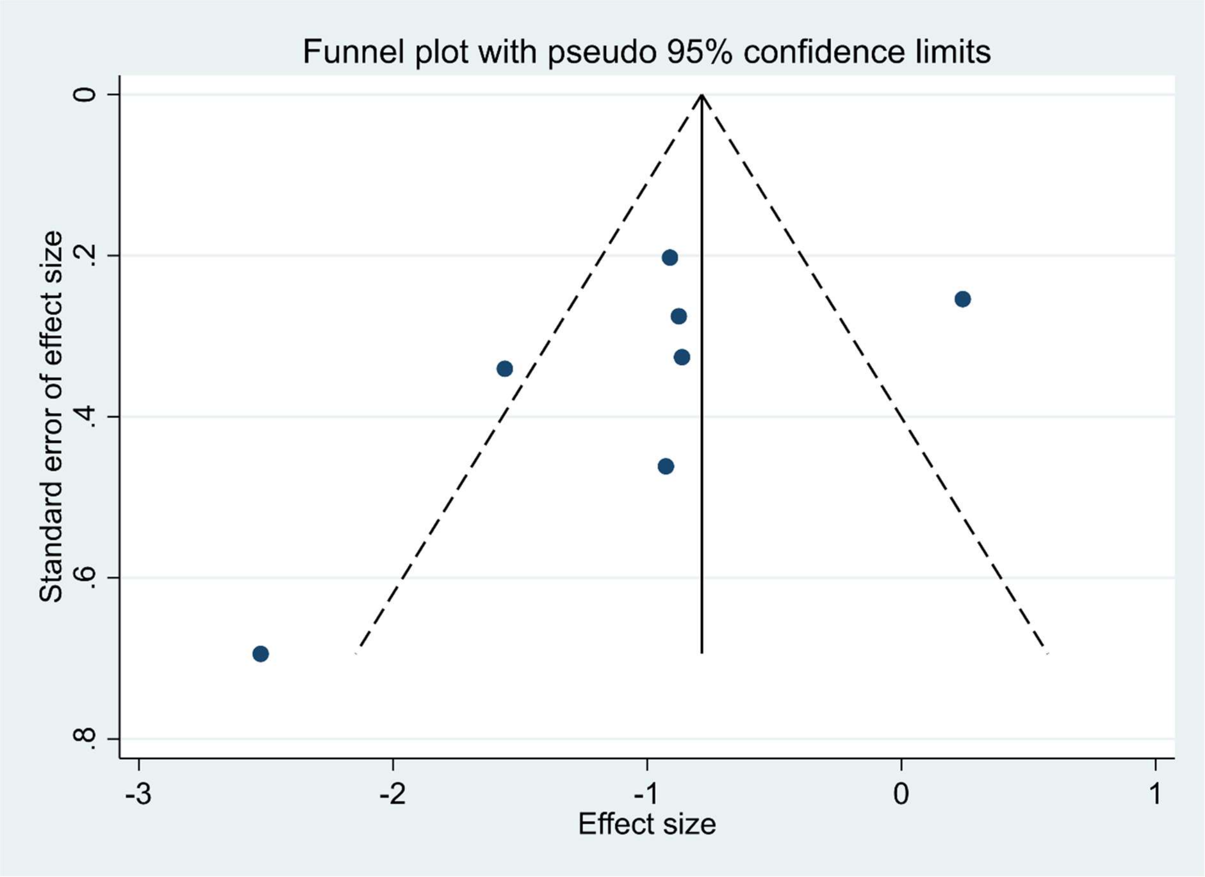

Supplementary Figure 5: A funnel plot showing the publication bias of studies on myoinositol/inositol intervention for preventing GDM.

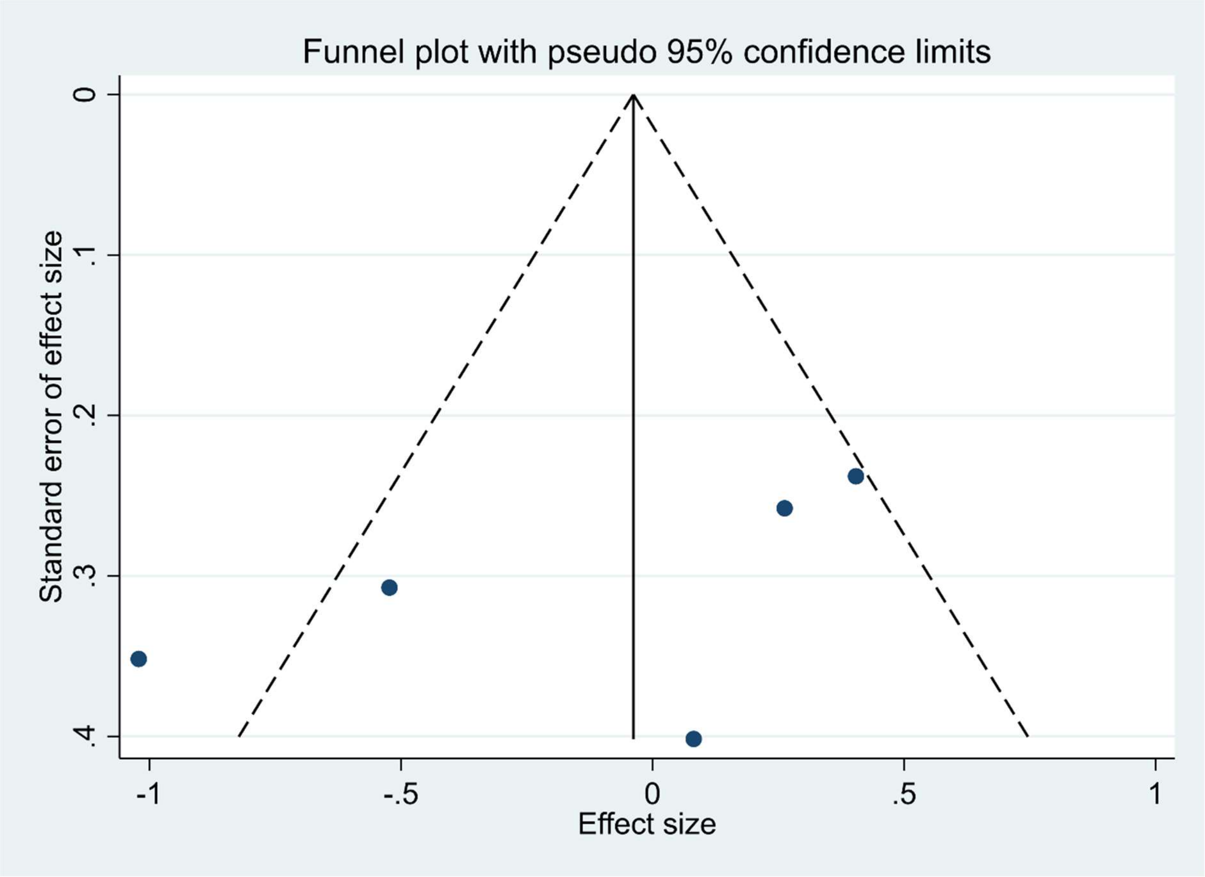

Supplementary Figure 6: A funnel plot showing the publication bias of studies on probiotics intervention for preventing GDM.
