# Supplementary material for: Participant characteristics in the prevention of gestational diabetes as evidence for precision medicine: a systematic review and meta-analysis
Source: Commun Med (Lond). 2023 Oct 5;3:137. doi: 10.1038/s43856-023-00366-x (PMC10551015; doi:10.1038/s43856-023-00366-x)
Supplement: Supplementary file 9 — Description of Supplementary Materials [file 43856_2023_366_MOESM9_ESM.docx]

**Description of Additional Supplementary Files**

**File name:** Supplementary Data 1

**Description:** Characteristics of included studies in this review

**File name**: Supplementary Data 2

**Description**: Subgroup analysis of dietary interventions for gestational diabetes prevention, by participant characteristics

**File name**: Supplementary Data 3.

**Description**: Meta-regression for gestational diabetes prevention, by participant characteristics

**File name**: Supplementary Data 4.

**Description**: Subgroup analysis of physical activity interventions for gestational diabetes prevention, by participant characteristics

**File name**: Supplementary Data 5.

**Description**: Subgroup analysis of combined diet and physical activity interventions for gestational diabetes prevention, by participant characteristics

**File name**: Supplementary Data 6.

**Description**: Subgroup analysis of metformin interventions for gestational diabetes prevention, by participant characteristics

**File name**: Supplementary Data 7.

**Description**: Subgroup analysis of myoinositol/inositol for gestational diabetes prevention, by participant characteristics
