# Supplementary material for: Participant characteristics in the prevention of gestational diabetes as evidence for precision medicine: a systematic review and meta-analysis
Source: Commun Med (Lond). 2023 Oct 5;3:137. doi: 10.1038/s43856-023-00366-x (PMC10551015; doi:10.1038/s43856-023-00366-x)
Supplement: Supplementary file 10 — Supplmentary Data 2 [file 43856_2023_366_MOESM10_ESM.docx]

Supplementary Data 2. Subgroup analysis of the effect of dietary interventions compared with control for gestational diabetes prevention, by participant characteristics

| Intervention type | The number of studies included | Risk ratio | Confidence interval | Heterogeneity (I^2^) (%) | p-value for subgroups | Weight |
| --- | --- | --- | --- | --- | --- | --- |
| Gestational week at baseline |  |  |  |  | 0.15 |  |
| Preconception | 2 | 0.78 | 0.57, 1.05 | 0 |  | 16.8 |
| <12 gestation weeks | 4 | 0.73 | 0.56, 0.95 | 38.7 |  | 34.3 |
| 13-17 gestation weeks | 4 | 0.73 | 0.57, 0.95 | 2.1 |  | 18.7 |
| >18 gestation weeks | 5 | 0.96 | 0.59, 1.54 | 60.0 |  | 26.8 |
| Unspecified | 2 | 0.25 | 0.09, 0.62 | 0 |  | 3.4 |
| BMI |  |  |  |  | 0.23 |  |
| Normal weight | 3 | 0.53 | 0.25, 1.12 | 65.5 |  | 13.07 |
| Overweight/obese | 4 | 0.60 | 0.29, 1.19 | 64.1 |  | 16.7 |
| Obese | 7 | 1.23 | 0.92, 1.78 | 0 |  | 21.5 |
| All BMIs | 8 | 0.72 | 0.6, 0.87 | 24.4 |  | 48.8 |
| Educational status |  |  |  |  | 0.16 |  |
| With tertiary level education | 4 | 0.96 | 0.74, 1.24 | 40.5 |  | 36.9 |
| Without tertiary level education | 4 | 0.74 | 0.53, 1.03 | 28.3 |  | 25.5 |
| Unspecified | 9 | 0.63 | 0.44, 0.91 | 46.8 |  | 37.5 |
| Employment status |  |  |  |  | 0.73 |  |
| Employed | 1 | 0.72 | 0.42, 1.21 | - |  | 7.1 |
| Unemployed | 2 | 0.65 | 0.39, 1.10 | 55.2 |  | 15.6 |
| Unspecified | 14 | 0.81 | 0.64, 1.01 | 51.0 |  | 77.5 |
| Hypertension at baseline |  |  |  |  | 0.34 |  |
| Without | 5 | 0.69 | 0.53, 0.90 | 0 |  | 25.3 |
| Unspecified | 12 | 0.82 | 0.65, 1.0 | 57.3 |  | 74.7 |
| Prediabetes at entry |  |  |  |  | 0.87 |  |
| Without | 5 | 0.69 | 0.46, 1.02 | 62.4 |  | 36.3 |
| Unspecified | 11 | 0.79 | 0.65, 0.98 | 27.1 |  | 63.7 |
| Parity |  |  |  |  | 0.23 |  |
| Nulliparous | 0 | - | - | - | - | - |
| Not nulliparous | 1 | 0.82 | 0.31, 2.19 | - |  | 2.9 |
| Mixed | 11 | 0.84 | 0.67, 1.06 | 58.3 |  | 75.1 |
| Unspecified | 5 | 0.60 | 0.45, 0.82 | 0 |  | 21.9 |
| Ethnicity |  |  |  |  | 0.12 |  |
| White | 3 | 1.23 | 0.79, 1.90 | 0 |  | 10.3 |
| Non-white | 5 | 0.86 | 0.61, 1.23 | 24.5 |  | 24.2 |
| Mixed | 3 | 0.65 | 0.47, 0.89 | 56.5 |  | 27.2 |
| Unspecified | 6 | 0.73 | 0.52, 1.03 | 54.6 |  | 38.3 |
| History of giving birth to large-for-age/macrocosmic infant |  |  |  |  | 0.25 |  |
| With | 1 | 0.82 | 0.31, 2.19 | - |  | 2.9 |
| Without | 1 | 0.47 | 0.25, 0.87 | - |  | 5.8 |
| Unspecified | 15 | 0.8 | 0.66, 0.98 | 47.5 |  | 91.2 |
| History of GDM |  |  |  |  | 0.21 |  |
| Without | 3 | 0.67 | 0.53, 0.83 | 0 |  | 21.3 |
| Unspecified | 14 | 0.82 | 0.65, 1.02 | 48.6 |  | 78.7 |
| History of HDP |  |  |  |  | 0.09 |  |
| Without | 1 | 0.47 | 0.25, 0.87 | - |  | 5.8 |
| Unspecified | 16 | 0.8 | 0.67, 0.97 | 43.8 |  | 94.2 |

GDM: gestational diabetes, BMI: body mass index, HDP: hypertensive disorders of pregnancy
